# Genome Analysis of *Clostridium difficile* PCR Ribotype 014 Lineage in Australian Pigs and Humans Reveals a Diverse Genetic Repertoire and Signatures of Long-Range Interspecies Transmission

**DOI:** 10.3389/fmicb.2016.02138

**Published:** 2017-01-11

**Authors:** Daniel R. Knight, Michele M. Squire, Deirdre A. Collins, Thomas V. Riley

**Affiliations:** ^1^Microbiology and Immunology, School of Pathology and Laboratory Medicine, The University of Western AustraliaNedlands, WA, Australia; ^2^Department of Population Health, School of Medical and Health Sciences, Edith Cowan UniversityJoondalup, WA, Australia; ^3^PathWest Laboratory Medicine, Department of Microbiology, Queen Elizabeth II Medical CentreNedlands, WA, Australia; ^4^Department of Medical and Molecular Sciences, School of Veterinary and Life Sciences, Murdoch UniversityMurdoch, WA, Australia

**Keywords:** CDI, phylogenomics, zoonosis, pan-genome, antimicrobial resistance, porcine, One Health

## Abstract

*Clostridium difficile* PCR ribotype (RT) 014 is well-established in both human and porcine populations in Australia, raising the possibility that *C. difficile* infection (CDI) may have a zoonotic or foodborne etiology. Here, whole genome sequencing and high-resolution core genome phylogenetics were performed on a contemporaneous collection of 40 Australian RT014 isolates of human and porcine origin. Phylogenies based on MLST (7 loci, STs 2, 13, and 49) and core orthologous genes (1260 loci) showed clustering of human and porcine strains indicative of very recent shared ancestry. Core genome single nucleotide variant (SNV) analysis found 42% of human strains showed a clonal relationship (separated by ≤2 SNVs in their core genome) with one or more porcine strains, consistent with recent inter-host transmission. Clones were spread over a vast geographic area with 50% of the human cases occurring without recent healthcare exposure. These findings suggest a persistent community reservoir with long-range dissemination, potentially due to agricultural recycling of piggery effluent. We also provide the first pan-genome analysis for this lineage, characterizing its resistome, prophage content, and *in silico* virulence potential. The RT014 is defined by a large “open” pan-genome (7587 genes) comprising a core genome of 2296 genes (30.3% of the total gene repertoire) and an accessory genome of 5291 genes. Antimicrobial resistance genotypes and phenotypes varied across host populations and ST lineages and were characterized by resistance to tetracycline [*tetM, tetA(P), tetB(P)* and *tetW*], clindamycin/erythromycin (*ermB*), and aminoglycosides (*aph3-III-Sat4A-ant6-Ia*). Resistance was mediated by clinically important mobile genetic elements, most notably Tn*6194* (harboring *ermB*) and a novel variant of Tn*5397* (harboring *tetM*). Numerous clinically important prophages (*Siphoviridae* and *Myoviridae*) were identified as well as an uncommon accessory gene regulator locus (*agr3*). Conservation in the pathogenicity locus and S-layer correlated with ST affiliation, further extending the concept of clonal *C. difficile* lineages. This study provides novel insights on the genetic variability and strain relatedness of *C. difficile* RT014, a lineage of emerging One Health importance. Ongoing molecular and genomic surveillance of strains in humans, animals, food, and the environment is imperative to identify opportunities to reduce the overall CDI burden.

## Introduction

*Clostridium difficile* is one of the most important causes of healthcare-associated infections worldwide, responsible for a quarter of all cases of infectious diarrhea and an array of serious complications including sepsis, toxic megacolon, and pseudomembranous colitis (Barbut and Petit, 2001). The last two decades have seen a paradigm shift in the epidemiology of *C. difficile* infection (CDI). The disease came to global prominence in the early 2000s due to the emergence and transcontinental spread of strains of a virulent lineage PCR ribotype (RT) 027 in the hospital environment (He et al., 2013). CDI reached an epidemic state in many developed countries with high incidence and severe disease in healthcare settings and in the community. In some regions of the world, including Australia, community-associated CDI (CA-CDI) now accounts for up to 25% of all cases (Slimings et al., 2014; Bloomfield and Riley, 2016).

The *C. difficile* genome (~4.3 Mbp) contains a high proportion of mobile genetic elements (11% in strain 630) and the species displays a large pan-genome estimated at over 9000 coding sequences (CDS), but remarkably shows an ultra-low level of genome conservation (as low as 16%; Sebaihia et al., 2006; Scaria et al., 2010; Forgetta et al., 2011; Treangen et al., 2014).

Increasingly, studies show that genetically diverse sources of *C. difficile* play a role in CDI transmission, challenging the assumption that CDI is primarily a healthcare-associated infection. In the 2013 landmark study of Eyre and colleagues, whole genome sequencing (WGS) of 957 isolates from 1250 patients with CDI at hospitals and in the community around Oxford, UK, found that 45% were genetically diverse (differing by >10 single nucleotide variants (SNV) in their core genome; Eyre et al., 2013). Over a third of these cases were erroneously linked by contact with another symptomatic case, staff contact or epidemiological type e.g., RT and sequence type (ST). Using an estimated molecular clock of ~1 SNV per-genome per-year the authors concluded that these isolates were genetically distinct from all other cases and likely originated from either asymptomatic carriage or a source other than the hospital environment (Eyre et al., 2013).

Possible reservoirs of CDI external to the healthcare system include wild animals, domesticated animals, performance animals, food, water, soil, farm animals, and their effluent or effluent by-products including compost (Hensgens et al., 2012; Squire et al., 2015; Bloomfield and Riley, 2016). *C. difficile* is particularly prevalent in production animals such as piglets and calves both in Australia (Knight et al., 2014, 2016) and other countries (Hensgens et al., 2012; Rodriguez et al., 2016). Furthermore, genetically similar strains of *C. difficile* (characterized by RT, MLST, MLVA, and WGS), particularly toxinotype V (RT078), have been recovered from humans, production animals and retail meat, fuelling speculation that CDI may have a foodborne or zoonotic etiology (Rupnik and Songer, 2010; Knetsch et al., 2014).

Five *C. difficile* evolutionary clades are currently recognized and are increasingly associated with specific continents; clade 1 (Europe), clade 2 (North America), clade 3 (potentially Africa), clade 4 (Asia), and clade 5 (Australia; Knight et al., 2015a). RT014 [toxinotype 0, North American Pulsotype (NAP) 4] is a highly successful clade 1 lineage of *C. difficile* consistently among the most common RTs causing CDI in Europe (Bauer et al., 2011; Freeman et al., 2014) and is predominant in some pediatric populations (Schwartz et al., 2014). RT014 is also currently the most prevalent RT in Australia, accounting for ~25% of CDI cases (Foster et al., 2014; Knight et al., 2015b; Collins et al., in press). In 2013, we conducted a prevalence study of *C. difficile* in 21 piggeries across five states in Australia and found RT014 to be the most prevalent RT in neonatal pigs, accounting for 23% (*n* = 26/154) of isolates (Knight et al., 2014). To date, assessment of the genetic overlap between these two populations has been limited to low-resolution genotyping tools and the analysis of small discrete regions of the *C. difficile* genome such as the 16S–23S rRNA intergenic spacer region (ISR) and the pathogenicity and binary toxin loci (Knight et al., 2015a). Based on RT and toxin gene profiles alone, one could suggest that zoonotic (animal to human) or anthroponotic (human to animal) transmission of RT014 is occurring.

In this study, to further define the extent of genetic overlap and detect evidence of potential transmission events, we performed WGS and high-resolution core genome phylogenetics on a contemporaneous collection of Australian RT014 isolates of human and porcine origin. In addition, the overall genetic repertoire of this RT014 strain collection was investigated by pan-genome analysis and characterization of the *C. difficile* RT014 resistome, prophage content and *in silico* virulence potential.

## Materials and methods

### Strain collection

A total of 40 *C. difficile* RT014 isolates from humans and pigs in Australia were included in this study. Porcine isolates (P1–P16) were sourced from pigs aged < 14 days from six piggeries (farms) during the period April 2012 and December 2012 (Knight et al., 2014). The farms were located in four states; Victoria [VIC; *n* = 7 isolates, *n* = 2 farms (MAR, BRM)], New South Wales [NSW; *n* = 4 isolates, *n* = 2 farms (YNG1, YNG2)], Queensland [QLD; *n* = 3 isolates, *n* = 1 farm (MDB)], and South Australia [SA; *n* = 2 isolates, *n* = 1 farm (SHK)]. All piglets, with the exception of P1–4 (farms YNG1, YNG2), were from farms with a history of idiopathic neonatal scouring.

Human isolates (H1–H24) were sourced from CDI patients as part of a national CDI surveillance program conducted between November 2012 and April 2013 (Collins et al., in press). Isolates originated from seven laboratories (sites) associated with tertiary hospitals in the same four states; VIC [*n* = 9 isolates, *n* = 2 sites (MLB, CLN)], NSW [*n* = 8 isolates, *n* = 3 sites (MQP, RWK, SLD)], QLD [*n* = 4 isolates, *n* = 1 site (TNG)] and SA [*n* = 3 isolates, *n* = 1 site (ADL)]. CDI cases were defined according to guidelines proposed by Cohen et al. (2010); 11 were identified as healthcare-associated CDI (HA-CDI), 11 as CA-CDI and two as indeterminate (INDET).

For comparative analysis, the genomes of previously sequenced clinical *C. difficile* RT014 strains from European studies; ATCC43600 (GenBank accession SRP044633; Knetsch et al., 2012), Ox1533 (ERS139376), Ox593 (ERS139417) and Ox1475 (ERS139420; Dingle et al., 2013) were included in all bioinformatics analyses, making a total of 44 genomes. Details of all isolates and genomes analyzed in this study are shown in Table 1.

**Table 1:** **Strain collection**.

**Strain ID**	**Host**	**Sample type**	**Country**	**Source**	**Farm/hospital^†^**	**CDI exposure**	**Date of isolation**	**ENA accession**
P1	Porcine	Piglet	Australia	NSW	YNG1	–	Apr-2012	ERS1078766
P2	Porcine	Piglet	Australia	NSW	YNG1	–	Apr-2012	ERS1078744
P3	Porcine	Piglet	Australia	NSW	YNG1	–	Apr-2012	ERS1078745
P4	Porcine	Piglet	Australia	NSW	YNG2	–	Apr-2012	ERS1078746
P5	Porcine	Piglet	Australia	VIC	MAR	–	Jul-2012	ERS1078769
P6	Porcine	Piglet	Australia	VIC	MAR	–	Apr-2012	ERS1078767
P7	Porcine	Piglet	Australia	VIC	MAR	–	Jul-2012	ERS1078770
P8	Porcine	Piglet	Australia	VIC	MAR	–	Jul-2012	ERS1078771
P9	Porcine	Piglet	Australia	VIC	MAR	–	Jul-2012	ERS1078772
P10	Porcine	Piglet	Australia	VIC	BRM	–	Aug-2012	ERS1078773
P11	Porcine	Piglet	Australia	VIC	BRM	–	Aug-2012	ERS1078774
P12	Porcine	Piglet	Australia	QLD	MDB	–	Dec-2012	ERS1078747
P13	Porcine	Piglet	Australia	QLD	MDB	–	Dec-2012	ERS1078748
P14	Porcine	Piglet	Australia	QLD	MDB	–	Dec-2012	ERS1078749
P15	Porcine	Piglet	Australia	SA	SHK	–	Jun-2012	ERS1078758
P16	Porcine	Piglet	Australia	SA	SHK	–	Jun-2012	ERS1078759
H1	Human	Adult-CDI	Australia	VIC	MQP	HA-CDI	Nov-2012	ERS1078762
H2	Human	Adult-CDI	Australia	NSW	MQP	CA-CDI	Nov-2012	ERS1078763
H3	Human	Adult-CDI	Australia	VIC	RWK	INDET	Dec-2012	ERS1078764
H4	Human	Adult-CDI	Australia	NSW	MQP	INDET	Dec-2012	ERS1078768
H5	Human	Adult-CDI	Australia	NSW	MQP	CA-CDI	Dec-2012	ERS1078750
H6	Human	Adult-CDI	Australia	NSW	SLD	HA-CDI	Mar-2013	ERS1078754
H7	Human	Adult-CDI	Australia	NSW	SLD	HA-CDI	Mar-2013	ERS1078755
H8	Human	Adult-CDI	Australia	NSW	SLD	HA-CDI	Mar-2013	ERS1078756
H9	Human	Adult-CDI	Australia	QLD	TNG	CA-CDI	Nov-2012	ERS1078760
H10	Human	Adult-CDI	Australia	QLD	TNG	CA-CDI	Nov-2012	ERS1078761
H11	Human	Adult-CDI	Australia	QLD	TNG	CA-CDI	Jan-2013	ERS1078751
H12	Human	Adult-CDI	Australia	QLD	TNG	CA-CDI	Jan-2013	ERS1078752
H13	Human	Adult-CDI	Australia	SA	ADL	HA-CDI	Dec-2012	ERS1078765
H14	Human	Adult-CDI	Australia	SA	ADL	CA-CDI	Mar-2013	ERS1078753
H15	Human	Adult-CDI	Australia	SA	ADL	CA-CDI	Apr-2013	ERS1078757
H16	Human	Adult-CDI	Australia	VIC	MLB	HA-CDI	Nov-2012	ERS1078775
H17	Human	Adult-CDI	Australia	VIC	MLB	HA-CDI^*^	Nov-2012	ERS1078776
H18	Human	Adult-CDI	Australia	VIC	MLB	HA-CDI^*^	Nov-2012	ERS1078777
H19	Human	Adult-CDI	Australia	VIC	MLB	HA-CDI	Nov-2012	ERS1078778
H20	Human	Adult-CDI	Australia	VIC	MLB	CA-CDI	Nov-2012	ERS1078779
H21	Human	Adult-CDI	Australia	VIC	CLN	CA-CDI	Nov-2012	ERS1078780
H22	Human	Adult-CDI	Australia	VIC	CLN	CA-CDI	Nov-2012	ERS1078781
H23	Human	Child-CDI	Australia	VIC	CLN	HA-CDI	Nov-2012	ERS1078782
H24	Human	Adult-CDI	Australia	VIC	CLN	HA-CDI	Nov-2012	ERS1078783
Ox1533	Human	Adult-CDI	UK	OXF	–	HA-CDI	Oct-2008	ERS139376^a^
Ox593	Human	Adult-CDI	UK	OXF	–	HA-CDI	Aug-2007	ERS139417^a^
Ox1475	Human	Adult-CDI	UK	OXF	–	HA-CDI	Sep-2008	ERS139420^a^
43600^‡^	Human	Adult-CDI	ECDC	ECDC	–	–	–	SRP044633^b^

*NSW, New South Wales; VIC, Victoria; SA, South Australia; QLD, Queensland; OXF, Oxford, United Kingdom; CDI, Clostridium difficile infection; CA-CDI, community-associated CDI; HA-CDI, healthcare-associated CDI; INDET, indeterminate*.

† *de-identified farms and hospital labs*.

*ECDC, European Centre for Disease Prevention and Control reference library*.

‡ *ATCC reference strain*.

* *Residential aged care facility (RACF) onset*.

*ENA, European Nucleotide Archive*.

a *Dingle et al. (2013)*.

b *Knetsch et al. (2012)*.

### Genomic DNA preparation and whole genome sequencing

*C. difficile* culture was performed as previously described (Knight et al., 2015b). After subculture on blood agar for 24 h, 1–3 colonies of each isolate were inoculated into pre-reduced brain-heart infusion broth containing 0.1% L-Cysteine and incubated anaerobically at 37°C overnight (~16 h). Cells were pelleted, resuspended in phosphate-buffered saline and genomic DNA was extracted using a Gentra Puregene Kit [Qiagen GmbH, Hilden, Germany]. Multiplexed paired-end (PE) sequencing libraries were generated using standard Nextera XT protocols [Illumina Inc., San Diego, CA, USA] and sequencing was completed on MiSeq and HiSeq 2500 platforms [Illumina], generating 250 and 100 bp PE reads, respectively. Sequencing yielded a median PE read count of 5,259,522 (99% ≥Q30), resulting in a theoretical fold coverage of 99X across all isolates. Fastq files were trimmed for quality and adapter content using Trimmomatic v0.33 (Bolger et al., 2014). Illumina PE reads have been submitted to the European Nucleotide Archive under study PRJEB12970 (sample accessions ERS1078744—ERS1078783).

### *In silico* multilocus sequence typing and antimicrobial resistance gene profiling

PE sequence reads were interrogated for multi-locus sequence type (MLST) and acquired antimicrobial resistance genes using pubMLST and ARG-ANNOT databases respectively, compiled within SRST2 v0.1.8 (Griffiths et al., 2010; Gupta et al., 2014; Inouye et al., 2014). A maximum-likelihood (ML) tree was generated from MUSCLE-aligned concatenated allele sequences (seven loci, 3501 bp) using PhyML v3.0 with an Hasegawa-Kishino-Yano (HKY) evolutionary model and 1000 random bootstrap replicates (Edgar, 2004; Guindon et al., 2009).

### *De novo* assembly and annotation

Trimmed reads were assembled *de novo* using SPAdes v3.6 (Bankevich et al., 2012) or in cases where contiguity was low, the A5 pipeline (Coil et al., 2015). ABACAS v1.3.1 (Assefa et al., 2009) was used to order and orientate contigs relative to the genome of reference strain CD630 (GenBank accession AM180355.1, ST54, clade 1) and GMcloser v1.3 (Kosugi et al., 2015) was used for gap closure and contig extension. Finally, *ab initio* annotation was performed using the rapid genome annotation pipeline Prokka v1.11 (Seemann, 2014). Annotated *C. difficile* assemblies are freely available at the online research data repository FigShare [https://figshare.com] using the following link http://dx.doi.org/10.6084/m9.figshare.4290266.

### Orthologous gene clustering and microevolutionary analysis

To identify a core set of orthologous genes for microevolutionary analysis, *de novo* assembled RT014 genomes were analyzed using three independent orthology-calling algorithms; COGtriangles (COG), OrthoMCL (OMCL), and bidirectional best-hit (BDBH), all implemented in the program GET_HOMOLOGUES v2.0.6 (Contreras-Moreira and Vinuesa, 2013), following the approaches of previous studies of the *C. difficile* core genome (Scaria et al., 2010; Forgetta et al., 2011; Treangen et al., 2014). To ensure confidence in clustering of homologous and not paralogous gene families the following stringent conditions were applied; (i) minimum BLAST pairwise alignment coverage of 90%, (ii) minimum BlastP sequence identity of 95%, (iii) *E*-value threshold of 1e^−10^, and (iv) inparalogs were excluded. Finally, an intersection of the results generated by all three algorithms was performed resulting in a consensus set of orthologous gene clusters. Gene-by-gene alignment was performed using MAFFT v2.273 (L-INS-I mode; Katoh et al., 2002). The resulting alignment was concatenated and used for ML inference in RAxML v7.0.4 with a general time reversible (GTR) model of evolution and GAMMA approximation for substitutional heterogeneity (Stamatakis, 2006).

Homologous recombination has the potential to distort bacterial phylogenies and can result in exaggerated branch lengths and an elevation of the evolutionary distance between strains (Knight et al., 2015a). In order to mitigate its effects, the RAxML best fit tree and MAFFT alignment were used as input for ClonalFrameML (v1.0) (Didelot and Wilson, 2015). ClonalFrameML simultaneously detects clusters of loci containing elevated densities of base substitutions, identifies them as recombination events and generates a final tree that has been corrected for recombination. Default parameters were used and the reliability of each node was supported by 1000 random bootstrap resamplings of the data. Trees were mid-point rooted and curated using FigTree v1.4.2 (Rambaut, 2007).

### Single nucleotide variant analysis

Short read mapping, variant calling, and filtering were performed using methods developed for transmission analysis of *Staphylococcus aureus* (Harris et al., 2010). The pipeline has since been developed and widely implemented in microevolutionary studies of *C. difficile* (Didelot et al., 2012; Eyre et al., 2013, 2015; Knetsch et al., 2014; Mac Aogáin et al., 2015; Stone et al., 2016).

Trimmed PE reads from each isolate were mapped to the finished reference genome CD630 using Smalt v0.7.6 (http://www.sanger.ac.uk/science/tools/smalt-0). Candidate core genome SNVs were identified across all mapped sites using a Bayesian statistical framework implemented by the algorithms *mpileup* and *view* within SAMtools v0.1.12–10 (Li et al., 2009). Using a combination of VCFtools v0.1.13 (Danecek et al., 2011), SnpEff v4.2 (Cingolani et al., 2012) and in-house Unix scripts, a series of stringent filtering steps was performed on the raw base calls to remove false positives and to extract only high quality *bona fide* variant sites for subsequent downstream analyses.

SNVs had to be of high quality (Phred-scaled QUAL score ≥200), supported by a read consensus of 75%, a minimum of five reads (including one in each direction) and SNVs were required to be homozygous under a diploid model (GT = 1/1). SNVs occurring in regions of unusual depth (>threshold of 3× median depth for that isolate) were not called. Indels were removed and SNVs were only called if they fell within unique (non-repetitive) regions of the reference chromosome, determined by constructing a mask of CD630 sequence regions with self-similarity (Morgulis et al., 2006). To alleviate the confounding effect of homologous recombination in the SNV data set we used Gubbins v1.4.5 (Croucher et al., 2015). Firstly, to generate the required input for Gubbins, consensus fasta files were produced for each sample with variant sites positioned on the CD630 backbone, resulting in a final pseudomolecule for each sample of 4,290,252 bp. Gubbins rapidly and iteratively scans the sequence alignment, identifying regions of heightened base substitution density. These putative recombination “hotspots” were then removed resulting in a final set of high quality concatenated SNVs in “clonal frame” (Didelot et al., 2012).

Finally, SNVs were annotated using SnpEff (Cingolani et al., 2012) and pairwise SNV differences (ΔSNVs) between all isolates was calculated using a custom python script kindly provided by David W. Eyre (University of Oxford). Using this approach an average of 93.5% of sites within the CD630 chromosome (4,012,699 bp) were mapped to a median depth of 98.4X. A final alignment of concatenated SNVs was used as input for RAxML as described above, except a CAT approximation for substitutional heterogeneity was used (Stamatakis, 2006).

### Comparative genomic analysis of transposons and prophage discovery

*De novo* assemblies were interrogated for the presence of transposons (Tns) using a custom sequence library comprising Tns previously identified in *C. difficile* and other related Firmicutes. The library included but was not limited to, Tn*916* (accession U09422), Tn*1549* (AF192329), Tn*4451* (U15027), Tn*4453a* (AF226276.1), Tn*5397* (AF333235.1), Tn*5398* (AF109075.2), Tn*6194* (HG475346.1), Tn*6215* (KC166248.1), Tn*6218* (HG002387.1), and Tn*B1230* (AJ222769.3). Genomes with matches to known Tns were manually investigated for the presence of signature genes and CDS, sequence homology and overall synteny. Comparative analysis was performed using MUMmer v3.0 (Kurtz et al., 2004), Blastn v2.3.0 (Altschul et al., 1990), Artemis (Carver et al., 2012), and Easyfig v2.1 (Sullivan et al., 2011). Plasmids were not specifically investigated in this study.

Predictions of prophage sequences within the RT014 genomes was investigated using PHASTER [http://phaster.ca/], a new implementation of the PHAST (PHAge Search Tool) web server (Arndt et al., 2016). Prophages were detected by querying of contigs against viral and prophage databases in Genbank and scored on the principle of completeness, i.e. the presence, quality and synteny of known phage genetic features including length, gene content, GC content, and attachment sites. Results were recorded as intact (scoring between 90 and 150), questionable (scoring between 60 and 90) and incomplete (scoring < 60) as previously described (Arndt et al., 2016).

### *In vitro* antimicrobial susceptibility testing

Minimum inhibitory concentrations (MIC) were determined for a panel of 16 antimicrobial agents against all RT014 isolates (*n* = 40) using CLSI agar dilution methodology as previously described (Knight et al., 2015b). The panel included vancomycin, metronidazole, fidaxomicin, rifaximin, clindamycin, erythromycin, amoxicillin-clavulanate, piperacillin-tazobactam, ceftriaxone, meropenem, moxifloxacin, tetracycline, trimethoprim, gentamicin, tobramycin, and spectinomycin. Where available, clinical breakpoints for antimicrobial agents are those based on recommendations of CLSI and EUCAST as previously detailed (Knight et al., 2015b). For fidaxomicin, a European Medical Agency proposed susceptible breakpoint of 1 mg/L was used (report WC500119707, http://www.ema.europa.eu/).

### Comparative analysis of virulence factors, conserved genes, and clinically relevant loci

To corroborate PCR toxin gene profiling results, genomes were screened for the presence and synteny of genes common to the Pathogenicity locus (PaLoc; *tcdR, tcdB, tcdE, tcdA*, and *tcdC*) and binary toxin locus (CdtLoc; *cdtR, cdtA, cdtB*). *De novo* assemblies were also submitted to the Bacterial Isolate Genome Sequence Database (BIGSdb) (Jolley and Maiden, 2010) for allelic characterization of clinically relevant loci including (i) *slpA* and the cell wall protein (cwp) gene cluster, (ii) the receptor binding domain (RBD) of *tcdB*, (iii) PaLoc negative regulator *tcdC*, (iv) RNA polymerase (*rpoB*), and (v) the quinolone resistance-determining regions (QRDR) of *gyrA* and *gyrB*.

We also investigated nucleotide sequence conservation in a set of 45 genes present in all RT014 genomes that are associated with virulence and host-pathogen interaction (see Section Results, **Table 4**). Sequences were aligned using MUSCLE and Neighbor-Joining (NJ) trees supported by 500 bootstrap replicates were generated in MEGA6 with evolutionary distances calculated using the Tajima-Nei model (Edgar, 2004; Tamura et al., 2013).

### Pan-genome estimation, regression analysis, and functional annotation

Analysis of the *C. difficile* RT014 pan, core, and accessory genome was performed using Roary v3.6.0 (Page et al., 2015) and PanGP v1.0.1 (Zhao et al., 2014). Roary was run with default parameters. PanGP was run using a distance guide (DG) subsampling algorithm with 100 replicates and 1000 permutations of genome order generating distribution plots of (i) total genes, (ii) conserved genes and (iii) new genes found upon progressive sampling of “*n*” genomes.

Definitions of the core and pan-genome and estimates of their respective size and trajectory were made using models and regression algorithms proposed by Tettelin and colleagues (Tettelin et al., 2005, 2008; Rasko et al., 2008), and used in previous *C. difficile* core and pan-genome studies (Scaria et al., 2010; Forgetta et al., 2011; Treangen et al., 2014). The curve fitting of the pan-genome was performed using a power-law regression model based on Heaps law [$y=A_{pan}x^{B_{pan}}+C_{pan}$] as previously described (Tettelin et al., 2008), where *y* denotes pan-genome size, *x* the genome number and *A*_*pan*_, *B*_*pan*_, and *C*_*pan*_ are fitting parameters. Here, *B*_*pan*_ is equivalent to the parameter γ used by Tettelin et al. in estimating the open or closed nature of a pan-genome (Tettelin et al., 2008). When 0 < *B*_*pan*_ < 1, the size of the pan-genome increases unboundedly with sequential addition of new genomes and can be considered open. Conversely, when *B*_*pan*_ < 0 or > 1 the pan-genome trajectory approaches a plateau as further genomes are added and can be considered closed. The curve fitting of core-genome was performed using an exponential regression model [$y=A_{core}e^{\left(B_{core}x\right)}+C_{core}$] (Rasko et al., 2008; Tettelin et al., 2008). New gene plots were derived from the pan-genome showing the number of new “strain-specific” genes contributing to the pan-genome per additional sequenced strain as a function of the number of strains.

Functional categorization of the RT014 proteome was performed by comparison of amino acid sequences from all CDS found in the pan-genome against the Kyoto Encyclopedia of Genes and Genomes (KEGG) database using the web tool blastKOALA (Kanehisa et al., 2016). Similar analysis was performed on CDS comprising the pan-genome of the human and porcine groups.

## Results

### *C. difficile* RT014 genome characteristics

Metrics and general features for 44 RT014 genomes evaluated in this study are presented in Table 2 and Supplementary Table 1. Variations in genome size and content were found across the RT014 genomes. Genomes ranged in size from ~4.0 to ~4.4 Mb, harboring between 3654 and 4248 CDS and an average of 53 tRNAs, 13 rRNAs, and 11 CRISPRs (Clustered regularly interspaced short palindromic repeats). Overall, CDS accounted for 79–87% of the average genome size (4.26 Mb) and GC percentage ranged between 28 and 30% (median 28.6).

**Table 2:** **Genome metric summary**.

**Metric**	**Median (Range)**
Genome size (Mbp)	4.26 (4.02–4.40)
GC%	28.64 (28.26–29.46)
N genes	3910 (3728–4305)
N CDS	3832 (3654–4248)
CDS length	768 (63–8877)
Total CDS length (Mbp)	3.49 (3.33–3.75)
Coding % (of genome)	82.40 (79.39–87.17)
Coding density (CDS/Mbp)	906 (869–979)
N tRNA	53 (32–77)
N rRNA	13 (7–26)
N CRISPRs	11 (8–19)
N Contigs	124 (27–422)
N50	100,109 (18,427–564,959)

*CRISPRs, Clustered regularly interspaced short palindromic repeats*.

### MLST

**Figure 1:**
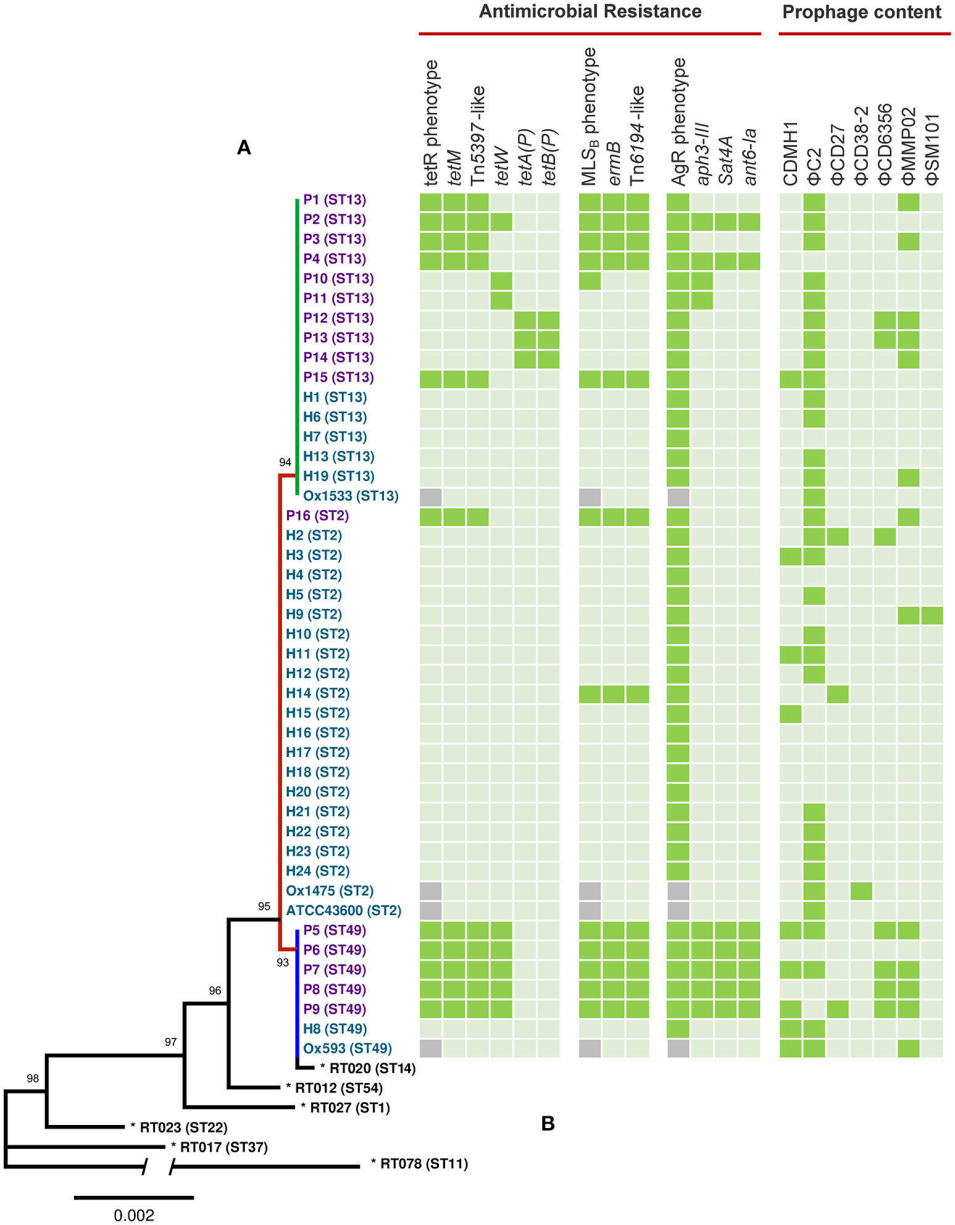
**MLST, antimicrobial resistance, and prophage analysis for ***C. difficile*** RT014 from humans and animals in Australia. (A)** Maximum likelihood MLST phylogeny. Scale shows the number of substitutions per site, based on concatenated MLST allele sequences (7 loci, 3501 bp). For global phylogenetic context, well-characterized representatives of MLST clades 1 (ST54, RT012), 2 (ST1, RT027), 3 (ST22, RT023), 4 (ST37, RT017), and 5 (ST11, RT078) are also shown (^*^). For comparative purposes ST14 (RT020), an RT often grouped with RT014 is also included. Tree is mid-point rooted and is supported by 1000 bootstrap replicates (only values >50 are shown). The branch depicting divergent ST 11 contains a break; the overall length of this branch is 0.0144. Branch and taxa coloring/labeling for RT014 strains; teal, human (H); purple, porcine (P); red, ST2 (*n* = 21); green, ST13 (*n* = 16); blue, ST49 (*n* = 7). **(B)** Heatmap visualizing the distribution of antimicrobial resistance elements, associated phenotypes and prophage content. Presence (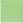), absence (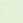), MICs were not determined for UK strains Ox1533, Ox1475, Ox1593, and ATCC43600 (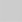). Some genomes harbored duplicate copies of prophages; P3 (2x ΦC2), P7 (2x ΦC2), P15 (3x ΦC2), H8 (2x ΦC2), H19 (2x ΦC2 and 2x ΦMMP02), and Ox1475 (2x ΦC2).

An MLST phylogeny for 44 *C. difficile* RT014 genomes is shown in Figure 1. RT014 was differentiated into three sequence types (STs): ST2 (H strains, *n* = 20, P strains, *n* = 1), ST13 (H strains, *n* = 6, P strains, *n* = 10), and ST49 (H strains, *n* = 2, P strains, *n* = 5). Notably, human and porcine populations were intermingled, particularly in the ST13 group. All STs display allelic conservation in five of the seven housekeeping genes (*adk, atpA, glyA, recA*, and *sodA*) but differed by single polymorphisms in *tpi* and *dxr* genes (data not shown).

### Phylogenetic analysis of core orthologous gene clusters

**Figure 2:**
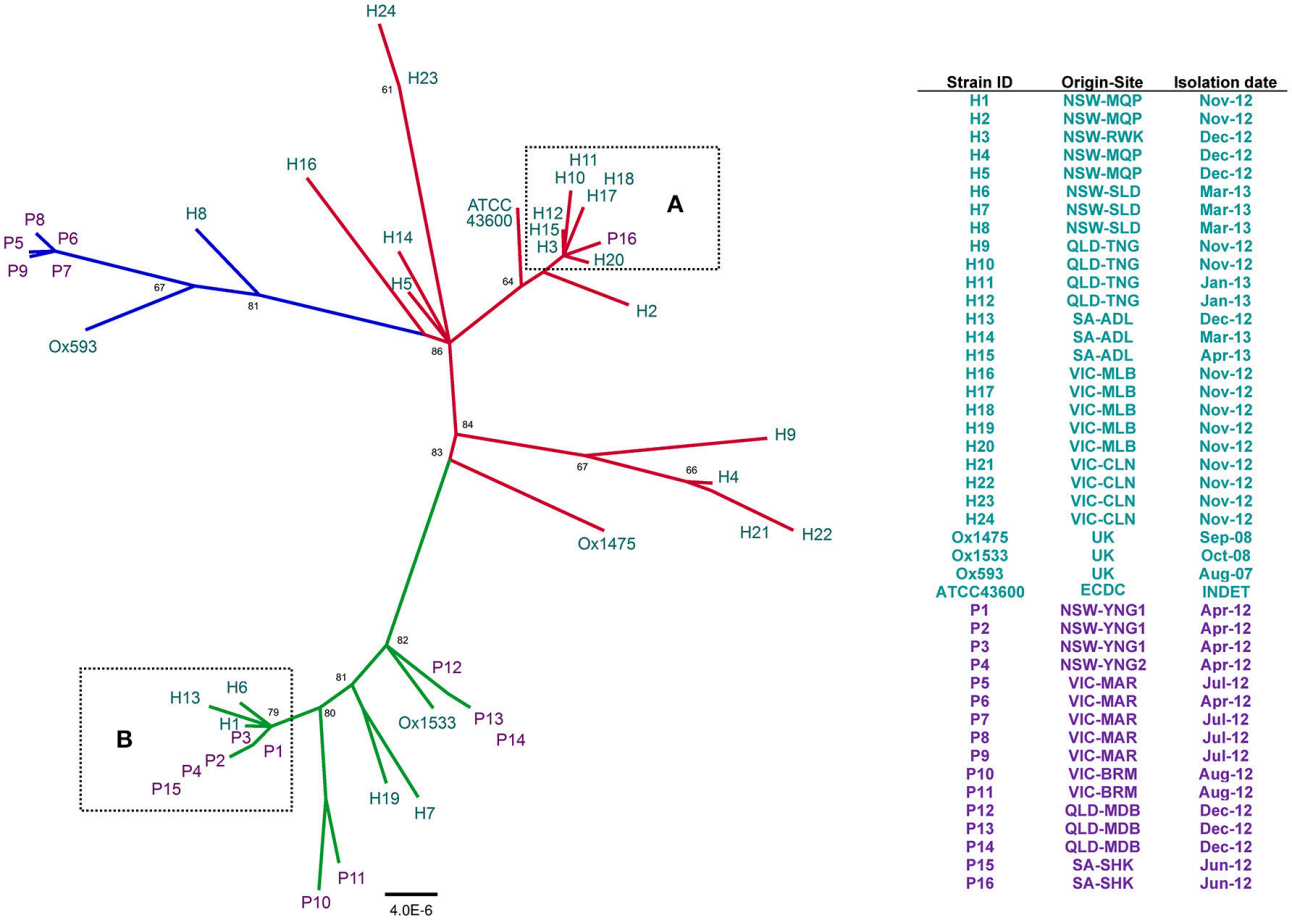
**Population structure of 44 ***C. difficile*** RT014 based on core orthologous genes**. ClonalframeML radial phylogram constructed from gene-by-gene alignment of 1260 orthologous gene clusters (1,019,160 bp). Tree is mid-point rooted and the nodes are supported by 1000 bootstrap replicates (only values >50 are shown). Scale bar represents the number of the number of substitutions per nucleotide site. Branch and taxa coloring/labeling for RT014 strains; teal, human (H); purple, porcine (P); red, ST2 (*n* = 21); green, ST13 (*n* = 16); blue, ST49 (*n* = 7). Boxes **(A,B)** indicate interspecies clustering in ST lineages 2 and 13, respectively. Legend shows corresponding information for strain ID, origin, site, and date of collection.

We employed a stringent consensus orthology-calling approach to identify a set of homologous genes present in all 44 RT014 strains that could be used for robust high-resolution phylogenetic analysis. A total of 1296, 1334, and 1296 orthologous gene clusters were identified by OMCL, COG, and BDBH algorithms, respectively (see Section Materials and Methods). An intersection of these three estimates yielded a robust consensus set of 1260 orthologous genes (1,019,160 bp) used for ML tree building with ClonalframeML. Microevolutionary analysis of 44 RT014 strains is shown in Figure 2. The 21 strains comprising the ST2 group were resolved into multiple strain clusters (Figure 2). Some strains that shared a common geographic and temporal relationship were located on distant parts of the phylogeny suggesting significant genetic heterogeneity e.g., H21–22 and H23–24 (all Nov-12, VIC-CLN). Conversely, some strains showed clustering despite an absence of a common geographic and temporal relationship e.g., H4 (NSW-MQP, Dec-12), H9 (QLD-TNG, Nov-12), and H21–22 (both VIC-CLN, Nov-12). Most notable was the clustering of eight human ST2 strains (H3, H10–12, H15, H17–18, and H20) with a single porcine strain (P16) (Figure 2, **Box A**). These strains originated from multiple states (NSW, QLD, SA, and VIC) and were collected over an 11-month period (Jun-12–Apr-13). These data suggest a very recent shared ancestry and possible long-range transmission events.

The 16 strains comprising the ST13 group were resolved into numerous distinct clusters (Figure 2). The phylogeny revealed a cluster of three human and five porcine strains (H1, H6, H13, P1–4, and P15; Figure 2, **Box B**). These strains originated from NSW and SA and were collected over a 12-month period (Apr-12–Mar-13), again suggesting long-range transmission but also short-range inter-farm transmission (P1–3 and P4). Two other clusters were exclusively of porcine origin: P12–14 (QLD-MDB, Dec-12) and P10–11 (VIC-BRM, Aug-12). A third cluster contained H7 (NSW-SLD, Mar-13) and H19 (VIC-MLB, Nov-12).

The seven strains comprising the ST49 group were differentiated into three distinct clusters, one containing all five porcine strains (P5–9) (Figure 2). The phylogenetic distance between P5–9 and the two human strains (H8 and Ox593) suggest a lack of recent common ancestry and limited genetic overlap. The four international strains (Ox1533, Ox1475, Ox593, and ATCC-43600) did not show significant clustering with any of the Australian RT014s (Figure 2).

### Single nucleotide variant analysis

SNV analysis provides ultra-fine scale resolution of bacterial populations and when interpreted in the context of a species molecular clock (a theoretical approximation of evolutionary change over time) is a powerful tool for identifying subtle genetic variability and signatures of clonal transmission (Didelot et al., 2012; Eyre and Walker, 2013). For *C. difficile*, a number of studies have calculated a fixed-rate molecular clock in the region of 1.47 × 10^−7^–5.33 × 10^−7^ mutations per site per-year, equating to 1–2 SNVs per-genome per-year (Didelot et al., 2012; Eyre et al., 2013; He et al., 2013; Knetsch et al., 2014). Therefore, a cut-off of 0–2 SNVs has been proposed as a signature of a recent clonal transmission event (Didelot et al., 2012; Knetsch et al., 2014; Mac Aogáin et al., 2015; Stone et al., 2016).

**Figure 3:**
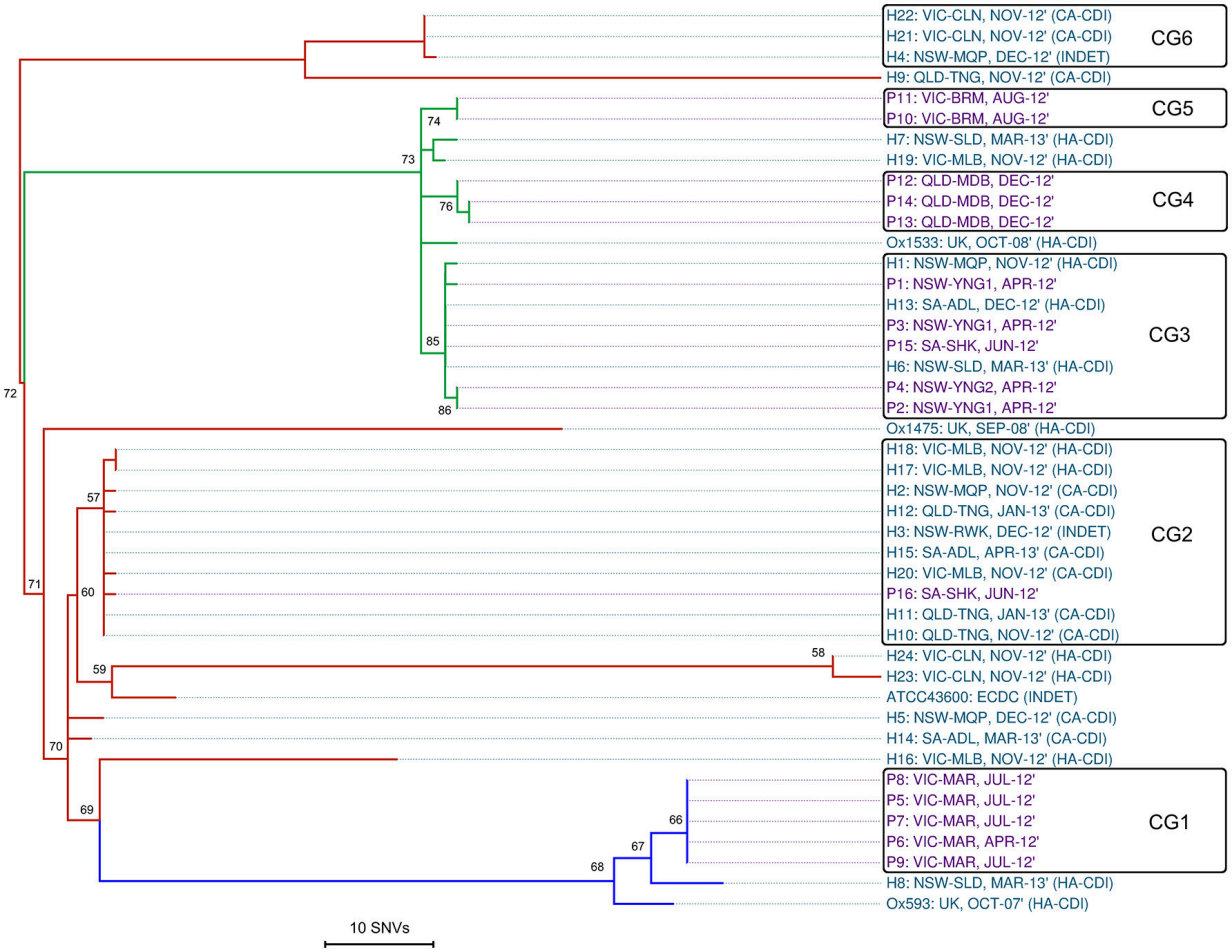
**Single nucleotide variant analysis of 44 ***C. difficile*** RT014**. Maximum-likelihood phylogeny based on non-recombinant SNVs (*n* = 1287) identified after mapping all sequence reads against the CD630 reference genome (accession AM180355, 4,290,252 bp). RAxML tree is mid-point rooted and is supported by 1000 non-parametric bootstrap replicates (only values >50 are shown). Branch and taxa coloring/labeling for RT014 strains; teal, human (H); purple, porcine (P); red, ST2 (*n* = 21); green, ST13 (*n* = 16); blue, ST49 (*n* = 7). Taxa labels include ID: ORIGIN-SITE, ISOLATION DATE, and ACQUISITION STATUS (if known). The black boxes indicate a clonal group (CG) where all isolates differ by no more than two SNVs (0–2). To enhance the visual resolution of the relative evolutionary distances (branch lengths/tips) between test genomes, CD630 was omitted from the final phylogeny (mean 1069 SNV differences from test genomes).

**Figure 4:**
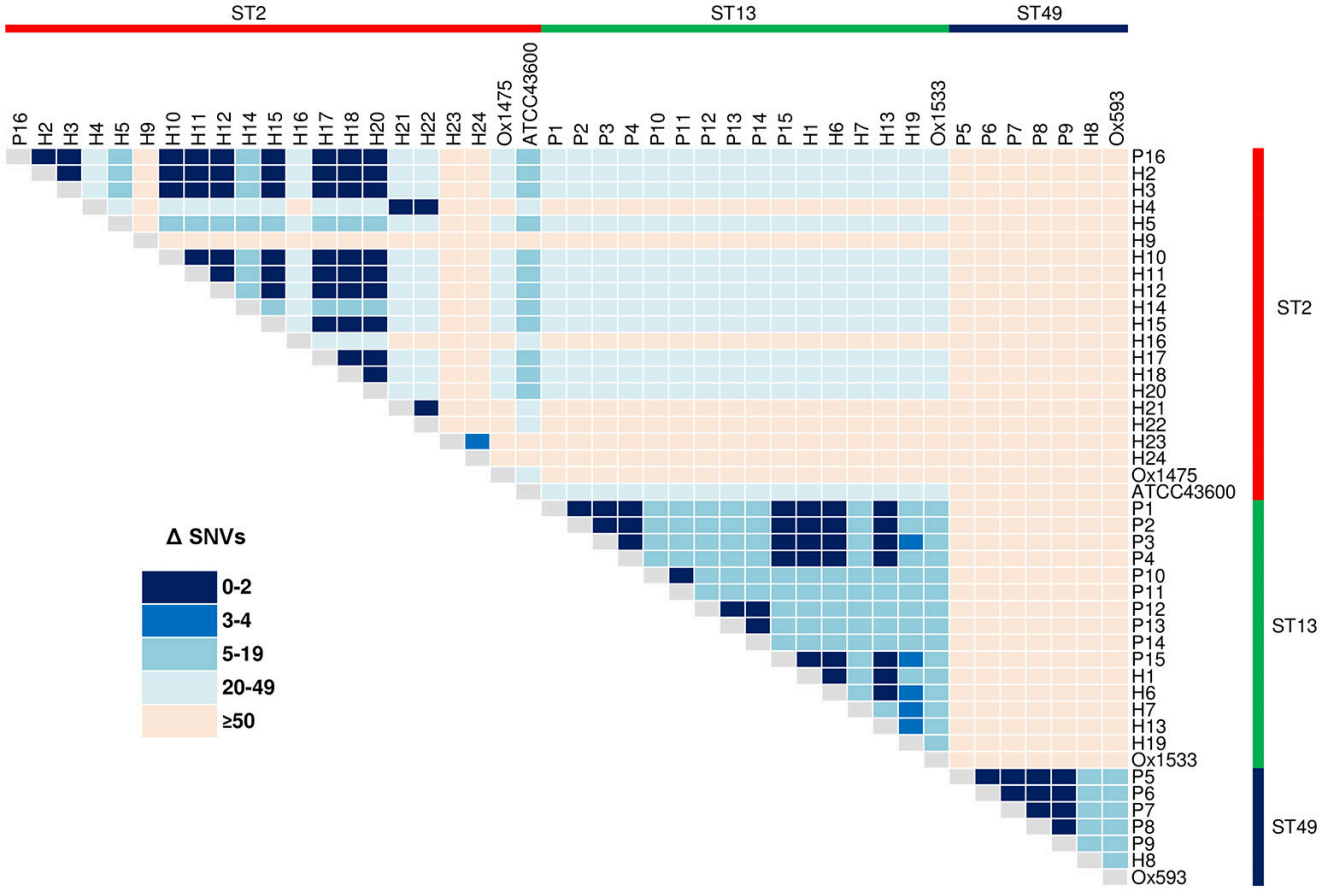
**Core genome SNV distances between 44 ***C. difficile*** RT014**. Heatmap of pairwise core genome SNV differences (Δ) between all 44 isolates, sorted by ST: red, ST2 (*n* = 21); green, ST13 (*n* = 16); and blue, ST49 (*n* = 7).

Reference mapping and a stringent filtering pipeline yielded 1287 high-quality *bona fide* SNVs across the 44-sample data set. Of these, 24.2% (*n* = 311) coded for non-synonymous gene changes, 69.5% (*n* = 894) coded for synonymous (silent) gene changes and 6.4% (*n* = 82) of sites were intergenic. A SNV based ML phylogeny of 44 RT014 in clonal frame is shown in Figure 3. A heatmap of pairwise SNV differences between all 44 genomes is shown in Figure 4. The ML tree is in agreement with the *de novo* phylogeny revealing (i) ST specific branches, (ii) a general absence of geographic clustering, and (iii) intermingling of human and animal strains in all three lineages. Applying a fixed-rate molecular clock of 1–2 SNVs per-genome per-year, six clonal groups (CGs) were identified, defined as strains differing by ≤ 2 SNVs in their core genome (Figure 3, CG1–6). Overall, 42% of human strains (12/28) showed a clonal relationship with one or more porcine strain (Figure 3). Based on geographic and temporal distributions, CGs 2 and 3 show signatures of long-range intra- and inter-species transmission events.

CG3 comprised three human and five porcine strains (all ST13) collected over a 12-month period (Apr-12–Mar-13). The strains originated from six distinct sites in NSW and SA separated by an average distance of 696 km, ranging from ~2 km (farms YNG1 and YNG2, both in NSW) to 1162 km (hospital sites ADL and SLD in SA and NSW). Within CG3, all human strains were classified as HA-CDI. Furthermore, we observed an apparent directionality in transmission events with all porcine strains isolated in Apr-12/Jun-12 prior to the human cases were in Nov-12, Dec-12, and Mar-13 (Figure 3).

CG2 was the largest CG, comprising nine human and a single porcine strain (all ST2). The majority (75%) of human strains were classified as CA-CDI. As with CG3, strains originated from sites distributed across a vast geographic area. The average distance between sites was 894 km with the largest distance (1597 km) found between sites TNG (QLD) and ADL (SA). Three human strains originated from a single site in QLD (TNG, Nov-12–Jan-13), three from a single lab in VIC (MLB, Nov-12), and two from different sites in NSW (MQP and RWK, Nov-12 and Dec-12, respectively; Figure 3). The single porcine strain (P16) originated from site SHK in SA and was collected in Jun-12 prior to all human strains. Overall, 50% of the human strains within CGs 2 and 3 originated from cases classified as CA-CDI whilst a seventh strain (H3) was categorized of indeterminate origin (Cohen et al., 2010), which in all probability, represents acquisition outside of the hospital system (onset > 4 weeks after leaving hospital).

The remaining four CGs showed clustering by host origin. Three CGs contained exclusively porcine strains indicating clonal populations in the respective piggeries: CG1 (*n* = 5, ST49, VIC-MAR, Apr/Jul-12); CG4 (*n* = 3, ST13, QLD-MDB, Dec-12), and CG5 (*n* = 2, ST13, VIC-BRM, Aug-12; Figure 3). CG6 contained three human ST2 strains collected in Nov-12 (*n* = 2, CA-CDI) and Dec-12 (*n* = 1, INDET) from geographically distinct sites in NSW and VIC (~711 km apart).

As observed in the *de novo* phylogeny, the ST2 group showed significant genetic diversity with strains H4, H9 and H21–22 present on a branch distinct from the other ST2 strains. The extent of variation in the ST2 group is illustrated by a distance of 133 SNVs between strains H9 and H23, which are found on most peripheral parts of the tree. Lastly, as seen in Figure 2, the four international RT014 strains were found to be distantly related to the Australian strains and an average of 1069 SNVs were found between RT014 strains and reference strain CD630 (ST54, RT012; data not shown).

### *In silico* antimicrobial resistance profiling

Sequenced RT014 genomes were surveyed for the presence of acquired antimicrobial resistance (AMR) genes. SRST2 identified 61 AMR genes with 60 (98.4%) found exclusively in porcine strains of ST lineages 13 and 49 (Figure 1). The methyltransferase gene *ermB* was found in 75% (*n* = 12/16) of porcine strains (ST2, *n* = 1/1; ST49, *n* = 5/5; ST13, *n* = 5/10) and a single human strain of ST2 (*p* < 0.005, χ^2^-test). A diverse collection of tetracycline resistance (tetR) genes was identified with a varied distribution in porcine strains of ST13 and ST49 but notably absent from human strains. Resistance elements *tetM, tetW, tetA(P)*, and *tetB(P)* were found in 68.8% (*n* = 11/16), 50.0% (*n* = 8/16), 18.8% (*n* = 3/16), and 18.8% (*n* = 3/16) of porcine strains, respectively (Figure 1).

Despite *C. difficile* being inherently resistant to aminoglycosides, 56.3% (*n* = 9/16) of porcine strains carried one or more genes belonging to an aminoglycoside-streptothricin resistance cassette (*aph3-III-Sat4A-ant6-Ia*). Seven porcine strains (five from ST49 and two from ST13) carried the complete cassette, whilst two (both ST49) retained only *aph3-III* (Figure 1). A single porcine strain (P12, ST13) harbored the lincomycin resistance gene, *lnuC* (Achard et al., 2005). Manual curation of the draft assemblies found all isolates harbored bacitracin (*uppP2*) and tellurium (*terD1*–*4*) resistance genes, the multidrug efflux resistance gene *cme* (Lebel et al., 2004), and a complete (cryptic) *vanG*_*Cd*_ operon (*vanR, vanS, vanG, vanY*, and *vanTG*; Ammam et al., 2013). All genomes were negative for resistance mutations within *rpoB* or the QRDR of *gyrA* and *gyrB*. Finally, as is characteristic for *C. difficile*, all strains harbored a gene encoding a β-lactamase inducing penicillin-binding protein (*blaR)*.

### *In vitro* antimicrobial susceptibility

Summary MIC data for 40 RT014 strains are presented in Table 3. Overall, rifaximin was the most active agent [geometric mean (GM) MIC = 0.004 mg/L, Kruskal-Wallis *H*-test *p* < *0.0001*], followed by fidaxomicin (GM MIC = 0.03 mg/L, *p* < *0.0001*), metronidazole (GM MIC = 0.33 mg/L, *p* < *0.0001*), and then vancomycin (GM MIC = 0.89 mg/L, *p* < *0.0001*). All isolates were fully susceptible, with no significant variation between human and porcine populations, to the first-line human therapies vancomycin, metronidazole, and fidaxomicin, as well as rifaximin, amoxicillin-clavulanate, moxifloxacin, trimethoprim, and piperacillin-tazobactam (Table 3). Comparison of human and porcine groups found differences in MIC for tetracycline (human GM MIC = 0.08 mg/L vs. porcine GM MIC = 5.85 mg/L, respectively; *p* < *0.0001*); erythromycin (GM MIC = 0.65 mg/L vs. GM MIC = 24.42 mg/L; *p* < *0.05*); clindamycin (GM MIC = 0.99 mg/L vs. GM MIC = 8.72 mg/L; *p* < *0.05*) and meropenem (GM MIC = 1.09 mg/L vs. GM MIC = 1.68 mg/L; *p* < *0.05*; Table 3).

**Table 3:** **Antimicrobial susceptibility data summary**.

**Agent**	**Human RT014 (*n* = 24)^†^**					**Porcine RT014 (*n* = 16)**					***P*-value^‡^**
	**Range (mg/L)**	**MIC50/90 (mg/L)**	**GM**	**%S**	**%NS**	**Range (mg/L)**	**MIC50/90 (mg/L)**	**GM**	**%S**	**%NS**	
VAN^a^	0.5–1	1/1	0.94	100	0	0.25–1	1/1	0.81	100	0	*p > 0.05*
MTZ^b^	0.12–0.5	0.5/0.5	0.36	100	0	0.12–0.5	0.25/0.5	0.28	100	0	*p > 0.05*
FDX^c^	0.004–0.12	0.03/0.06	0.03	100	0	0.004–0.12	0.06/0.06	0.03	100	0	*p > 0.05*
RFX^d^	0.002–0.008	0.004/0.008	0.004	100	0	0.002–0.015	0.004/0.008	0.004	100	0	*p > 0.05*
AMC^b^	0.12–0.5	0.25/0.5	0.23	100	0	0.12–0.5	0.25/0.5	0.25	100	0	*p > 0.05*
CLI^b^	0.12–8	2/4	0.99	88	12	0.25–>32	>32/>32	8.72	31	69	***p < 0.05***
ERY^b^	0.06–>256	0.5/4	0.65	96	4	0.12–>256	>256/>256	24.42	31	69	***p < 0.05***
CRO^b^	4–>128	16/32	13.85	79	21	8–32	16/32	16.71	81	19	*p > 0.05*
MEM^b^	0.5–2	1/2	1.09	100	0	0.5–2	1/2	1.68	100	0	***p < 0.05***
MXF^b^	0.5–2	1/2	1.22	100	0	0.5/1	1/1	0.84	100	0	***p < 0.05***
TET^b^	0.06–0.25	0.06/0.12	0.08	100	0	0.06–32	32/32	5.85	31	69	***p < 0.0001***
TZP^b^	0.5–8	4/8	3.89	100	0	0.5–8	4/8	4.18	100	0	*p > 0.05*
TMP	8–64	32/64	28.51	NR	NR	16–64	32/64	33.42	NR	NR	*p > 0.05*
GEN	16–64	16/64	20.16	NR	NR	16–32	16/32	19.03	NR	NR	*p > 0.05*
TOB	16–128	32/128	31.09	NR	NR	16–32	16/32	22.63	NR	NR	*p > 0.05*
SPC	16–128	32/128	57.02	NR	NR	32–128	32/128	47.26	NR	NR	*p > 0.05*

*VAN, vancomycin; MTZ, metronidazole; FDX, fidaxomicin; RFX, rifaximin; AMC, amoxicillin-clavulanate; CLI, clindamycin; ERY, erythromycin; CRO, ceftriaxone; MEM, meropenem; MXF, moxifloxacin; TET, tetracycline; TZP, piperacillin-tazobactam; TMP, trimethoprim, GEN, gentamicin; TOB, tobramycin; SPC, spectinomycin; S, susceptible; NS non-susceptible (intermediate and resistant breakpoints)*.

a *Breakpoint for VAN is recommended by EUCAST and is based on epidemiological cut-off values that distinguish “WT” isolates from those with reduced susceptibility*.

b *Breakpoints are those recommended for anaerobes by CLSI*.

c *Proposed susceptible breakpoint of 1 mg/L as recommended by EMA (report WC500119707, http://www.ema.europa.eu/)*.

d *Resistance (≥32 mg/L) is as described by O'Connor et al. (2008)*.

*^NR^, No breakpoints are currently available for trimethoprim, gentamicin, tobramycin, or spectinomycin*.

*GM, Geometric mean*.

‡ *Kruskal-Wallis H-test*.

† *testing not performed on UK isolates*.

*Values in bold are statistically significant at the 95% confidence level*.

*In vitro* antimicrobial activity for tetracycline, erythromycin, and clindamycin were largely congruent with the results of resistance gene profiling, with markedly different susceptibility profiles observed for human and porcine populations (Table 3). Overall, 32.5% of isolates, predominantly of porcine origin (H, *n* = 1/24, P, *n* = 12/16; *p* < *0.005*, χ^2^-test), presented an MLS_B_ phenotype. Of these, 92.3% (*n* = 12/13) harbored a concordant genotype (*ermB*), whilst a single strain (P10) was negative for *ermB*. Overall, 27.5% of isolates, exclusively of porcine origin (H, *n* = 0/24, P, *n* = 11/16) presented a tetR phenotype. Overall concordance with genotype was only 69% (*n* = 11/16) as several strains harboring only *tetW* (*n* = 2) or *tetA(P)* and *tetB(P)* did not show resistance *in vitro*. As expected, all isolates showed high MICs to the aminoglycosides gentamicin, tobramycin and spectinomycin, irrespective of harboring *aph3-III, Sat4A*, or *ant6-Ia*.

### Genomic context for antimicrobial resistance

**Figure 5:**
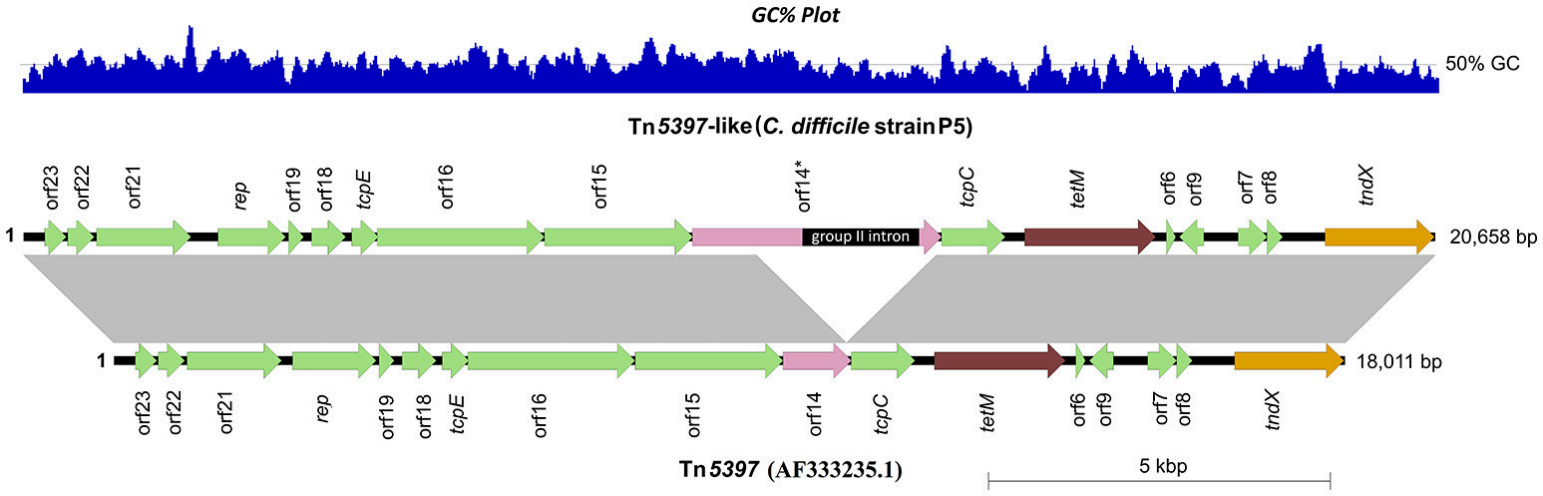
**Genetic organization of Tn***5397*** in RT014 genomes**. A representative Tn*5397*-like element from porcine strain P5 is shown compared to conjugative transposon Tn*5397* (AF333235.1). Arrows indicate open reading frames (ORFs) and direction of transcription. Characteristic features *tetM* (tetracycline resistance gene), *tndX* (site-specific recombinase gene) and ORF 14 are shown in brown, orange and pink respectively, with the remaining ORFs shown in green. ORF 14 is significantly truncated in P5 and completely lacks the 1831 bp group II intron (ORF 14^*^), a characteristic feature of Tn*5397*. Figure prepared using Easyfig (Sullivan et al., 2011). Gray vertical blocks between sequences indicate regions of homology (Blast nucleotide identity, >99%). Overall size (and GC%) of elements in P5 and AF333235.1 are 18,011 bp (38.5%) and 20,658 bp (38.4%), respectively.

To provide a genomic context for antimicrobial resistance, draft genomes were screened for the presence of Tns. A summary of identified Tns is shown in Figure 1. All 11 *tetM* positive isolates harbored identical elements showing >99% sequence identity and near perfect synteny with Tn*5397* (accession AF333235.1), the primary *tetM* encoding conjugative transposon found in *C. difficile* (Figure 5; Mullany et al., 2015). The archetypal Tn*5397* element is 20,658 bp in length and possesses three characteristic features: *tndX* (a serine recombinase used for excision and integration), *tetM* and ORF 14^*^ which is interrupted by a 1831 bp group II intron (Roberts and Mullany, 2011). The RT014 Tn*5397*-like elements showed conservation in 16 of 17 ORFs present in Tn*5397*. ORF 14 was truncated by 2648 bp and did not contain the classical Tn*5397* group II intron (Figure 5). The variant ORF 14 encodes a 333 amino acid (aa) product with 100% identity to a peptidase of the N1pC/P60 superfamily of peptidoglycan hydrolytic enzymes and is present in several Firmicute genera (accession WP_002324551.1).

In *C. perfringens, tetA(P)* and *tetB(P)* genes are carried on plasmid pCW3 and found complemented and overlapping by 22 bp. In RT014 strains P12–14, *tetA(P)* and *tetB(P)* were found in the same configuration only no discernible plasmid or Tn could be identified. The nucleotide sequences for *tetA(P)* was only a 92% match for *C. perfringens* (accession L20800) but a 100% match to *C. septicum* (AB054982) and *Turicibacter* sp. (CP013476.1). The nucleotide sequences for *tetB(P)* was a 99% match for *C. perfringens* (L20800) and *Turicibacter* sp. (CP013476.1).

We were not able to identify any discernible transposon upon detailed genomic analysis of the eight strains harboring *tetW*. All *tetW* genes in this population were identical and shared 100% sequence identity with *tetW* of transposon Tn*B1230* originating from the ruminant anaerobe *Butyrivibrio fibrisolvens* (accession AJ222769.3; Supplementary Image 1). The genes comprising the aminoglycoside-streptothricin cassette (*aph3-III-Sat4A-ant6-Ia*) were not found on any known mobilizable element. However, for the seven strains harboring all three genes, we were able to identify a 7272 bp region sharing 99% sequence identity with a multi-drug resistance cassette found in a strain of *Erysipelothrix rhusiopathiae* isolated from swine (accession KP339868.1). The genomic origin for the *aph3-III* genes found in P10 and P11 were also matches for other *E. rhusiopathiae* genomes.

All 12 *ermB* positive strains harbored elements resembling the uncommon conjugative transposon Tn*6194* (HG475346.1). We found that this 28 kbp element was often fragmented in the RT014 genomes. Therefore, we identified Tn*6194* on the presence of 35 characteristic CDS including the following defining genetic features: (i) a single copy of *ermB* (unlike Tn*5398* which has two), (ii) excision module comprising integrase (*int*, 1446 bp) and excisionase (*xis*, 258 bp) genes, (iii) toxin/antitoxin genes, and (iv) 3′ cell surface protein (3045 bp).

### Prophage discovery

A total of 73 intact, 85 questionable, and 223 incomplete prophages were identified in the 44 RT014 genomes. A summary of the distribution and genetic features of intact prophages are shown in Figure 1 and Supplementary Table 2, respectively. The 73 intact prophages were made up of seven different prophage “types” with a varied distribution across all host populations and ST lineages. Clostridial prophage ΦC2 was most commonly found (*n* = 38) followed by ΦMMP02 (*n* = 14), CDMH1 (*n* = 9), ΦCD6356 (*n* = 7), ΦCD27 (*n* = 3), ΦCD38-2 (*n* = 1), and ΦSM101 (*n* = 1) with some strains possessing multiple copies of some types, particularly ΦC2 (Figure 1; Supplementary Table 2). The mean number of intact prophages per-genome for human and porcine populations was 1.29 and 2.31, respectively (*p* = 0.48, *T*-test). Prophage size ranged between 12.2 and 108.2 kb in length (median of ~50.5 kb). GC content of the prophages ranged between 26.7 and 37.4% (median of 29.4%), which is comparable to the average GC content for the *C. difficile* host (28.6%).

### Presence and sequence conservation of genes associated with virulence and host-pathogen interaction

Isolates had previously been characterized by PCR as positive for the major virulence factors toxin A (*tcdA*^+^) and toxin B (*tcdB*^+^), but negative for binary toxin genes (*cdtA/B*^−^; Knight et al., 2014; Collins et al., in press). *In silico* analysis corroborated these results, confirming all strains harbored genes common to the typical RT014 PaLoc (*tcdR, tcdB, tcdE, tcdA*, and wildtype *tcdC*) and CdtLoc (*cdtR and cdtA/B* pseudogenes). Comparative and phylogenetic analysis of *tcdA* was not attempted due to the difficulties in sequencing repetitive stretches of DNA found within the CROP domain of *tcdA* (Kurka et al., 2014).

We also investigated nucleotide sequence conservation in a set of 45 genes present in all RT014 genomes. The genes included 14 highly conserved “phylogenetic marker” genes previously described by Kurka et al. (2014) and 31 additional genes associated with the bacterial cell wall (*n* = 10), antimicrobial resistance (*n* = 7), *C. difficile* toxins (*n* = 5), quorum sensing (*n* = 3), motility (*n* = 2), sporulation (*n* = 2) and other functions (*n* = 2). Details of the 45 genes, their products, and the results of the sequence conservation analysis are shown in Table 4. Overall, 68.9% (*n* = 31) of the 45 analyzed genes showed 100% nucleotide conservation across all 44 RT014 genomes, irrespective of host species or ST lineage (Table 4). The remaining 14 genes (*atpA, rpoA, rpoB, rpoC, blaR, tcdR, tcdB, tcdC, slpA, cwp66, cwp2, cwp11, cwp25*, and *agrB*) showed variations in their nucleotide sequences (range 72.0–99.9%) across the RT014 genomes. Phylogenies for each of the aforementioned genes are shown in Supplementary Image 2, trees A–N.

**Table 4:** **Sequence conservation analysis**.

**Gene**	**Product**	**Locus tag**	**nt**		**aa**		**Gene phylogeny**
			**Length (bp)**	**% ID**	**length (aa)**	**% ID**	
**PHYLOGENETIC MARKER GENES ANALYZED IN Kurka et al. (****2014****)**							
*atpA(V)*	V-type ATP synthase subunit Alpha	CD630_29560	1779	100.0	592	100.0	–
*atpA*	ATPase subunit Alpha	CD630_34700	1503	99.7	500	100.0	SI 2A
*atpD*	V-type ATP synthase subunit D	CD630_29540	699	100.0	222	100.0	–
*atpB*	ATPase subunit Beta	CD630_29550	1374	100.0	457	100.0	–
*rpoA*	RNA polymerase A	CD630_00980	948	99.8	315	100.0	SI 2B
*rpoB*	RNA polymerase B	CD630_00660	3717	99.9	1238	99.9	SI 2C
*rpoC*	RNA polymerase C	CD630_00670	3486	99.9	1161	100.0	SI 2D
*gryA*	Gyrase A	CD630_00060	2427	100.0	807	100.0	–
*gyrB*	Gyrase B	CD630_00050	1902	100.0	633	100.0	–
*infB*	Translation initiation factor IF-2	CD630_13090	1941	100.0	646	100.0	–
*recA*	Recombinase A	CD630_13280	1047	100.0	348	100.0	–
*dnaK*	Heatshock protein 70	CD630_24610	1848	100.0	615	100.0	–
*groEL*	Heatshock protein 60	CD630_01740	1629	100.0	542	100.0	–
*fusA*	Elongation factor G	CD630_00700	2067	100.0	688	100.0	–
**MOTILITY**							
*fliD*	agellar hook-associated cap protein	CD630_02370	24	100.0	507	100.0	–
*fliC*	Flagellin C	CD630_02390	873	100.0	290	100.0	–
**SPORULATION**							
*spo0A*	Stage 0 sporulation protein A	CD630_12140	810	100.0	274	100.0	–
*sodA*	Spore coat protein-superoxide dismutase	CD630_16310	705	100.0	234	100.0	–
**ANTIMICROBIAL RESISTANCE**							
*blaR*	x003B2;-lactamase inducing penicillin binding protein	CD630_04700	00	98.7	599	97.8	SI 2E
*cme*	Multidrug resistance transporter protein	CD630_31980	1251	100.0	416	100.0	–
*vanR*	vanGcd: response regulator	CD630_16240	702	100.0	233	100.0	–
*vanS*	vanGcd: sensor histadine kinase	CD630_16250	1143	100.0	380	100.0	–
*vanG*	vanGcd: D-alanine–D-alanine ligase	CD630_16260	1099	100.0	366	100.0	–
*vanY*	vanGcd: D-alanyl–D-alanine decarboxypeptidase	CD630_16270	807	100.0	268	100.0	–
*vanTG*	vanGcd: alanine racemase	CD630_16280	2139	100.0	712	100.0	–
**TOXIN GENES [PATHOGENICITY LOCUS (PaLoc) AND BINARY TOXIN LOCUS (CdtLoc)]**							
*tcdR*	PaLoc: RNA polymerase sigma factor	CD630_06590	555	99.3	184	98.9	SI 2F
*tcdB*	PaLoc: cytotoxin B	CD630_06600	7101	99.9	2366	99.8	SI 2G
*tcdE*	PaLoc: holin-like pore forming protein	CD630_06610	501	100.0	166	100.0	–
*tcdC*	PaLoc: negative regulator	CD630_06640	699	99.9	232	99.6	SI 2H
*cdtR*	CdtLoc: binary toxin regulator	CD630_26030	747	100.0	248	100.0	–
**CELL WALL (S-LAYER AND *cwp* CLUSTER)**							
*slpA*	S-layer protein	CD630_27930	2199-2304	78.1	720-768	65.3	SI 2I
*sec2A*	Cell surface secretory translocase subunit	CD630_27920	2346	100.0	781	100.0	–
*cwp84*	Cell surface binding protein 84	CD630_27870	2412	100.0	803	100.0	–
*cwp66*	Cell surface binding protein 66	CD630_27890	1833	72.0	611	63.0	SI 2J
*cwp2*	Cell surface binding protein 2	CD630_27910	1872	90.4	623	89.2	SI 2K
*CD2790*	Putative LmbE-like deacetylase	CD630_27900	708	100.0	234	100.0	–
*cwp5*	Cell surface binding protein 5	CD630_27860	1578	100.0	525	100.0	–
*cwp11*	Cell surface binding protein 11	CD630_27950	1602	98.9	533	99.2	SI 2L
*cwp13*	Cell surface binding protein 13	CD630_17510	2388	100.0	795	100.0	–
*cwp25*	Cell surface binding protein 25	CD630_27910	942	99.6	313	99.7	SI 2M
**QUORUM SENSING (*agr* LOCUS)**							
*agrC*	Sensor kinase	CDR20291_02638	1380	100.0	459	100.0	–
*agrB*	Accessory gene regulator	CD630_27500	579	99.8	192	99.5	SI 2N
*agrD*	Cyclic autoinducer peptide	CD630_27491	147	100.0	48	100.0	–
**OTHER**							
*fbpA*	Fibronectin-binding protein A	CD630_25920	1776	100.0	591	100.0	–
*gluD*	NAD-specific glutamate dehydrogenase	CD630_01790	1266	100.0	411	100.0	–

*nt, nucleotide; aa, amino acid*.

*Locus tag, systematic gene identifier in sequenced genomes*.

*CD630, RT012 reference strain (Genbank AM180355)*.

*CDR20291, RT027 reference strain (Genbank FN545816)*.

*SI, Supplementary Image*.

Notably, variations in nucleotide sequence for some genes were congruent with one or more ST lineages. For example, there were two distinct and conserved clusters corresponding to ST lineages 49 and 2/13 for each of the genes encoding RNA polymerases (*rpoA, rpoB, rpoC*; Supplementary Image 2, trees B–D). For *blaR*, three distinct clusters were identified corresponding to STs 2, 13, and 49 with a single divergent sequence seen for strain H9 (Supplementary Image 2, tree E). Similarly, for *atpA*, three distinct sequences were found, largely congruent with ST lineage (Supplementary Image 2, tree A).

Three genes within the PaLoc showed sequence divergence across the data set (*tcdR, tcdB*, and *tcdC*; Supplementary Image 2, trees F–H). Three main clusters, highly congruent with the three ST lineages were found for *tcdR*, a positive regulator of toxin expression. The majority (91%) of strains shared an identical *tcdC* sequence, with four human ST2 strains (H4, H9, H21, and H22) showing identical but divergent sequences. For *tcdB*, two conserved clusters were identified (ST13 and STs 2/49). As with *tcdC*, strains H4, H9, H21, and H22 possessed distinct *tcdB* sequences. Inspection of the 597 bp C-terminus RBD found that all RT014 *tcdB* were identical (allele type three according to the scheme of Dingle et al., 2011).

*C. difficile* genes involved in the production and regulation of the bacterial surface layer (S-layer) are co-located within a 36.6 kb cassette known as the cell wall protein (cwp) gene cluster. According to the scheme of Dingle et al. (2013), we found that with the exception of strain Ox1475 (detailed below), all RT014 strains were S-layer cassette type variant 10, harboring identical allele types for the major *cwp* genes *CD2790* (allele 7), *cwp2* (allele 8), *cwp66* (allele 9), *cwp84* (allele 12), and *secA2* (allele 8). There was significant sequence divergence in the principal cwp gene *slpA*, encoding the major S-layer precursor protein and immunodominant antigen slpA (Dingle et al., 2013). A total of four *slpA* allele types were identified showing broad congruence with ST lineage: allele type 7 (*n* = 8, human/animal STs 49/2), allele type 9 (*n* = 29, human/animal STs 13/2), allele type 41 (*n* = 6, human/animal ST13) and allele type 241 (*n* = 1, strain Ox1475, ST2; Supplementary Image 2, tree I). We found evolution in *slpA* occurred under purifying selection (Tajima's D, *p* < *0.001*; Nei-Gojobori *Z*-test, *p* < *0.001*). Strain Ox1475 also showed divergent sequences for other slpA locus genes *cwp2, cwp11*, and *cwp66* (Supplementary Image 2, trees J–L) and two distinct variant groups were found for *cwp25* corresponding to mixtures of STs 2/49 and 2/13 (Supplementary Image 2, tree M).

Finally, we found that all RT014 genomes harbored an uncommon *agr* locus type (type *agr3*) comprising syntenic *agrC, agrB* and *agrD* genes. Within *agr3*, sequence variation was only observed in the *agrB* gene (encoding a quorum sensing peptide) with two separate groups corresponding to STs 13 and 2/49 (Supplementary Image 2, tree N).

### Pan-genome and proteome analysis

To explore the entire genomic repertoire of the *C. difficile* RT014 population, estimates of the pan, core and accessory genome were generated. The core genome is defined as orthologous loci conserved across the whole data set, i.e., genes present once in every isolate. The accessory genome contains partially shared and strain-specific genes, and the pan-genome encompasses the full complement of genes (Tettelin et al., 2005).

**Figure 6:**
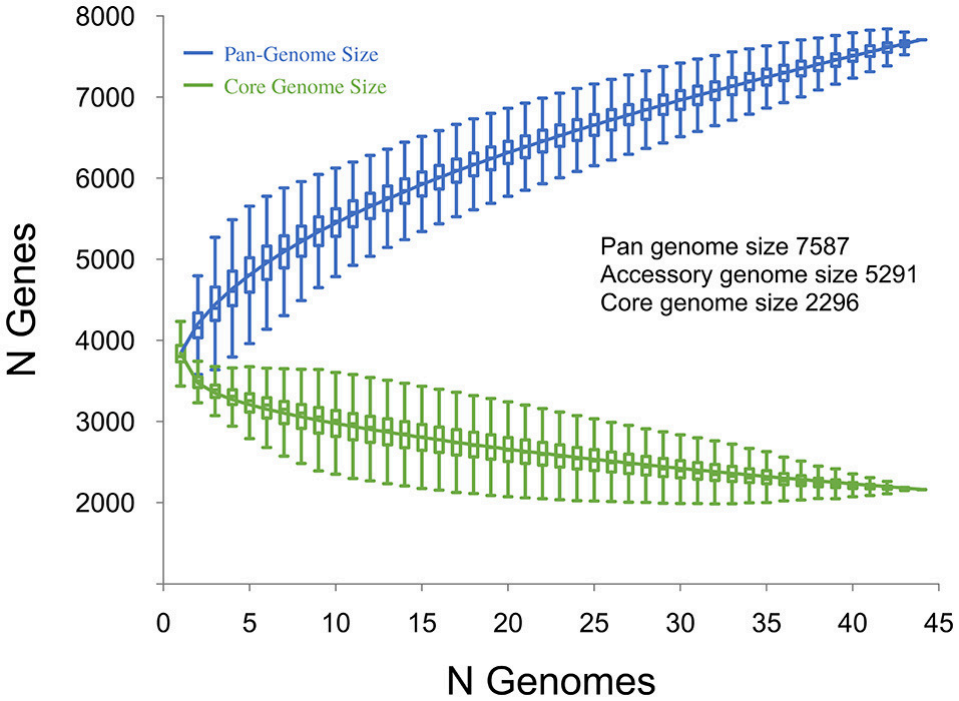
**Core and pan-genome analysis for 44 ***C. difficile*** RT014 genomes**. The total number of genes in the pan (blue) and core (green) genomes are plotted as a function of the number of genomes sequentially added (*n* = 44). Box plots indicate 25th and 75th percentiles with medians shown as horizontal lines and whiskers set at 10th and 90th percentiles. For the pan-genome, the continuous curve (blue) shows the fit (*r*^2^ = 0.999) of the power-law regression model. The pan-genome size is calculated at 7587 genes at *n* = 44 and displays characteristics of an open genome: (i) the trajectory of the pan-genome increases unboundedly as the number of genomes are added and (ii) *Bpan* (≈ γ, Tettelin et al., 2008) was estimated as 0.43. For the core genome, the continuous curve (green) shows the fit (*r*^2^ = 0.979) of the exponential regression model. The number of core genes converges to 2296 at *n* = 44, accounting for 30.3% of the total gene repertoire.

**Figure 7:**
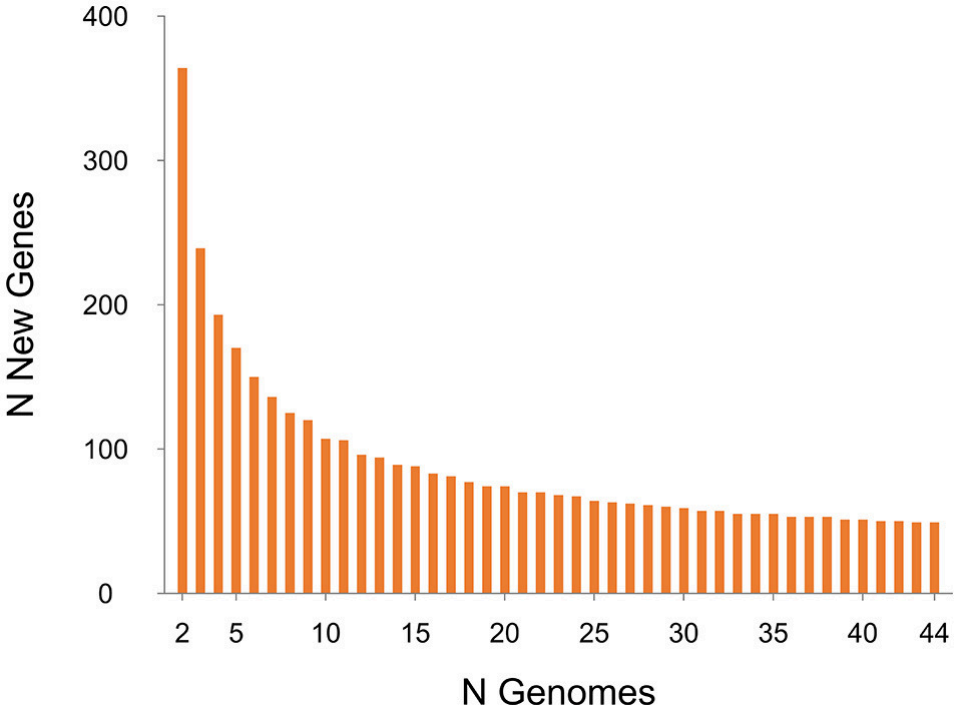
**Strain-specific gene analysis for 44 ***C. difficile*** RT014 genomes**. A plot of the number of new “strain-specific” genes contributing to the gene pool per additional sequenced strain as a function of the number of strains (*n* = 44). Consistent with an open pan-genome, the number of new genes does not converge to zero upon sequencing of additional genomes (at *n* = 44, an average of 48 new genes are contributed to the gene pool).

Plots visualizing the number of total genes, shared genes and distinct new genes as a function of the number of sequenced genomes are shown in Figure 6. The RT014 pan-genome comprised a total of 7587 genes, whilst the core and accessory genomes were 2296 and 5291 genes, respectively. The RT014 pan-genome shows characteristics of an “open” pan-genome (Tettelin et al., 2005). As depicted in Figure 6, the size of the pan-genome increases unboundedly with progressive sampling of new genomes. At *n* = 44, the pan-genome has already exceeded double the average number of genes found in a single RT014 genome (3832) and the plot is yet to reach a plateau indicating more sequenced strains are needed to capture the complete gene complement. Moreover, as shown in Figure 7, the number of new genes does not converge to zero upon sequencing of new strains (at *n* = 44, an average of 48 new genes are contributed to the gene pool). Supporting these observations, analysis of the pan-genome curve using a power-law regression model found the pan-genome is certainly open (*Bpan* = 0.43).

The core genome curve depicts a trend of core genome size contraction with progressive addition of sequential genomes (Figure 6). Exponential regression analysis shows the core genome decreases steadily with each sampled genome, converging at 2296 genes at *n* = 44 but does not reach a plateau. The core genome accounts for 30.3% of the total gene repertoire and 57.8% of an average RT014 genome CDS (range 50.1–60.63). Some studies report results in the context of a relaxed or soft-core genome, defined as those genes present in only 90 or 95% of strains (Ozer et al., 2014; Vernikos et al., 2015). We estimated the *C. difficile* RT014 soft-core genome to be 3322 and 3150 genes, respectively. Analysis of the accessory genome for this data set identified a collection of strain-specific genes also known as singletons (41.1%, *n* = 2169). Of these, over 850 were annotated with hypothetical or putative gene functions, many of phage origin (data not shown). Finally, we compared the pan-genomes of human and porcine RT014 groups (Supplementary Images 3, 4). The estimated pan, core and accessory genome sizes for 28 human strains and 16 porcine strains are 6278, 2935 and 3343, and 5688, 2546, and 3142 respectively.

**Figure 8:**
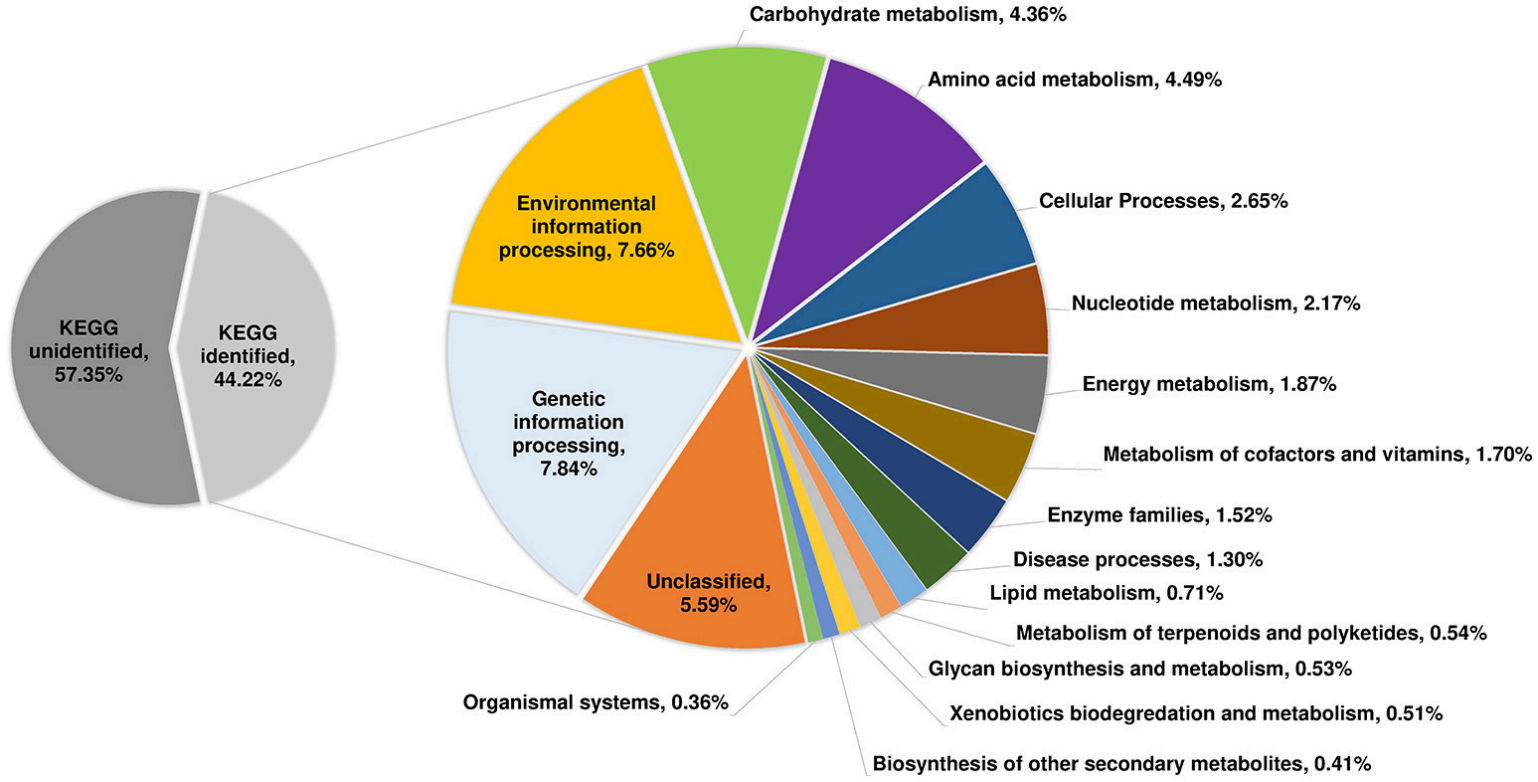
**Proteome analysis for 44 ***C. difficile*** RT014 genomes**. Functional proteomic characterization of CDS identified in the *C. difficile* RT014 pan-genome (*n* = 44). A total of 3355 CDS (44.22%) were classified by KEGG. CDS categorized as “unclassified” were identified by but no functional classification currently exists.

Proteomic analysis of the pan-genome was also performed in which a single representative sequence from each gene cluster (*n* = 7587) was interrogated against the KEGG database (Figure 8). Overall, 44.2% (*n* = 3355) of the predicted CDS were assigned to a functional category by KEGG. The functional categories with the largest number of assigned CDS are genetic information processing (7.84%) and environmental information processing (7.66%). Together, 17.3% of CDS belong to varied metabolism-based categories. Near identical proteomic profiles were obtained for human and porcine groups (≤ 0.75% difference in any of the 17 categories; Supplementary Image 5). A large proportion of CDS (~55%) were unidentified by KEGG, a result corroborated by an alternative database, the RAST Server (Aziz et al., 2008; data not shown). This suggests that the biological and physiological function of a large proportion of the *C. difficile* RT014 pan-genome/gene pool remains to be experimentally verified.

## Discussion

### *C. difficile* RT014 genome characteristics

RT014 is a highly successful lineage of *C. difficile* and among the most common RTs causing CDI in healthcare systems throughout the developed world (Bauer et al., 2011; Freeman et al., 2014; Schwartz et al., 2014; Lessa et al., 2015). We have previously reported that RT014 has established significant reservoirs in both human and porcine populations in Australia (Foster et al., 2014; Knight et al., 2014, 2015b; Collins et al., in press). These findings, together with a notable increase in CA-CDI in Australia, particularly in populations not considered to be at risk of CDI, have fuelled speculation that zoonotic transmission is a potential source of human infection (Slimings et al., 2014; Squire et al., 2015; Bloomfield and Riley, 2016). Using WGS and high-resolution core genome phylogenetics we describe for the first time the evolutionary relationships and extent of genetic overlap between strains of RT014 sourced from human and pigs in Australia. Furthermore, we provide characterization of the RT014 resistome, prophage content and virulence potential as well as the first pan-genome analysis for this important lineage.

### Microevolutionary analysis reveals signatures of long-range intra- and inter-species transmission

*In silico* MLST differentiated the 44 RT014 strain population into three STs (2, 13, and 49), all found within MLST clade 1 and all containing both human and animal strains. MLST is limited in genetic resolution as it focuses on just 0.1% of a typical 4.3 Mb *C. difficile* genome (7 genes, 3501 bp). Phylogenies based on the alignment of large sets of orthologous genes and on SNVs in the core genome provide ultra-fine scale resolution of *C. difficile* populations (Dingle et al., 2011; Eyre et al., 2013). We utilized both approaches, which were in agreement in identifying two defining features of the RT014 population structure.

Firstly, there was a general absence of geographical and temporal clustering for human strains and some porcine strains, indicating diversity exists between strains that are indistinguishable by RT. This is particularly evident for the isolates from MQP and SLD (NSW) and MLB and CLN (VIC) that were dispersed throughout the phylogeny (Figure 3). Secondly, there were multiple instances of human and porcine strains, some originating from Australian towns separated by thousands of kilometers and collected many months apart, that were either indistinguishable (0 SNVs) or very closely related (1–2 SNVs) in their core genome. Together, these data strongly suggest that over an extended period there has been frequent, long-range transmission of *C. difficile* RT014 between pigs and humans in Australia. Overall, SNV analysis substantiated inter-species transmission events for 42% of human strains and 37.5% of porcine strains. Strains belonging to the two interspecies clonal groups (CG2 and CG3) were isolated over long time periods; 11- and 12-months, respectively. In each case, porcine strains were collected several months prior to the corresponding human clone, possibly demonstrating a directionality and therefore evidence of zoonotic transmission. Moreover, 50% of the human strains within CGs 2 and 3 originated from cases classified as CA-CDI, which represents acquisition outside of the hospital system (onset > 4 weeks after leaving hospital).

The exact mode of transmission between pigs and humans remains unclear. In one study, the authors suggested that individuals working in pig husbandry or living in (or visiting) areas with a high density of pigs are at increased risk for acquiring *C. difficile* due to exposure to pig feces (Knetsch et al., 2014). To our knowledge, none of the human cases were linked to livestock occupations, or lived or worked close to piggeries; however, the transmission may have occurred via an asymptomatic intermediate (Durham et al., 2016). The finding that over half of the clonal cases of CDI occurred without recent healthcare exposure, and were separated by vast geographic distances, suggest a persistent community reservoir. A similar observation was made for a 2010–2012 outbreak of infection with *C. difficile* RT244 in Australia (Eyre et al., 2015).

Outside Australia, studies show retail meat, salads and vegetables are contaminated with *C. difficile* spores (Rupnik and Songer, 2010). Within Australia, two livestock/agricultural practices have been identified which could present significant risk for CA-CDI: (i) slaughtering of neonatal animals destined for human consumption, and (ii) the recycling of effluent to agriculture and compost manufacture with dissemination of contaminated vegetables and compost in the community setting (Squire and Riley, 2013; Squire et al., 2015).

Porcine CDI is almost exclusively a disease of neonates with *C. difficile* prevalence highest during the first 2 weeks of life, resulting in extensive spore contamination of the farrowing environment (Squire and Riley, 2013). However, unlike the Australian dairy industry where there remains a consumer demand for neonatal veal products (Knight et al., 2013, 2016), suckling age piglets are not slaughtered for meat on a large scale and are unlikely to contribute to a persistent or substantial community reservoir.

There are opportunities for long range *C. difficile* spore dissemination and contamination of food destined for human consumption including airborne transmission (Keessen et al., 2011) and avian, rodent, or arthropod vectors (Burt et al., 2012). In Australia, compost manufactured from pig feces and piggery effluent pond sludge is widely available for retail sale. Treated effluent pond wastewater is applied to land used for agriculture or pasture, or recycled for use within the facility. There is abundant evidence that *C. difficile* is found in treated biosolids and effluent (Viau and Peccia, 2009; Romano et al., 2012; Xu et al., 2014), including piggery effluent and wastewater treated in an on-site ponding system (Squire et al., 2011). *C. difficile* also survives land application of biosolids (Xu et al., 2016). Windrow composting reduces *C. difficile* spore load in biosolids but this is dependent on the endogenous strain and strict adherence to best-practice composting methodology (Xu et al., 2016). In Australia, *C. difficile* has been isolated from 20% (14/71) of vegetables grown in soil enriched with organic material and obtained from 11 diverse retailers. This comprised 5% (1/19) of carrots, 6% (1/18) of onions, 22% (4/18) of beetroot, and 50% (8/16) of potatoes (Lim et al., manuscript in preparation). This is a higher prevalence than that reported in studies using a similar methodology in the USA (0% in root vegetables, 2.4% in other vegetables; Rodriguez-Palacios et al., 2014) and Canada (4.5%; Metcalf et al., 2010).

Notably, we found a small number of instances of clonal transmission between piggeries in different states. Live animals, including suckling age piglets, are routinely moved between farms, sale yards, breeding centers, and abattoirs, and could contribute to the long-range dissemination of spores. Sow movement poses a particular risk, as they are housed on-farm in environments heavily contaminated with *C. difficile*. Unsurprisingly, *C. difficile* spores can be isolated from the feces and skin of healthy sows (Hopman et al., 2011).

There are several limitations to this analysis. The number of isolates investigated (*n* = 40) is low relative to this RTs contribution to human CDI and its prevalence in pig herds in Australia. Greater numbers of isolates from piggeries and from regional and tertiary hospitals would enhance our understanding of the complex transmission dynamics in these populations. We acknowledge that we did not include samples from food or piggery workers, which may have provided additional information about transmission chains and risk for consumers. Finally, the *C. difficile* molecular clock used to assess potential transmissions is an approximation based on within-host variation and the assumption of a constant rate of evolution. It does not account for the genetically quiescent nature of *C. difficile* spores and may underestimate the evolutionary distance between strains (Didelot et al., 2012; Eyre et al., 2013).

### *C. difficile* RT014 harbors a diverse repertoire of antimicrobial resistance genes and mobile genetic elements of clinical importance

Antimicrobial resistance plays a central role in driving epidemiological changes in *C. difficile* populations, a phenomenon exemplified by the emergence and global dissemination of fluoroquinolone-resistant epidemic RT027 (He et al., 2013). In this study, we found all Australian RT014 isolates susceptible to first-line human CDI therapies vancomycin, metronidazole and fidaxomicin, as well as rifaximin, amoxicillin-clavulanate, meropenem, moxifloxacin, piperacillin-tazobactam, and trimethoprim. These data are consistent with our earlier study (Knight et al., 2015b) and a large multi-site European study (Freeman et al., 2014). Acquired antimicrobial resistance in the RT014 population was limited to clindamycin, erythromycin, and tetracycline and mediated by clinically important mobile genetic elements.

Clindamycin exposure is recognized as a specific risk factor for CDI and clindamycin-resistant clinical strains of *C. difficile* are common throughout Europe, Asia, North America, Australia, and the Middle East (Knight et al., 2015b; Spigaglia, 2016). Clindamycin-resistant isolates usually show resistance to macrolide antimicrobials such as erythromycin (MLS_B_ phenotype) and resistance is most often mediated by ermB methylation of bacterial 23S rRNA (Spigaglia, 2016). In this study, 75% of porcine strains and a single human strain presented a MLS_B_ phenotype, all but one of which carried the *ermB* gene on a conjugative transposon, Tn*6194*. To our knowledge, this element has not been isolated from clinical strains in Australia, or from animals elsewhere in the world. Tn*6194* is the most common *ermB*-containing element in European clinical isolates, particularly epidemic RTs 027, 001, and 017 (Spigaglia, 2016). Moreover, this element is recognized as one of the defining genetic features of the epidemic RT027 sublineage FQR1 which disseminated and caused outbreaks with high mortality in North America and sporadic cases in Asia (He et al., 2013). Furthermore, genetic studies show this Tn is fully mobilisable with the capability of both intra-species transfer to different *C. difficile* RTs and inter-species transfer to *E. faecalis* (Wasels et al., 2014).

Despite a broad spectrum of activity against both Gram-positive and negative bacteria including many gut anaerobes, tetracycline exposure is considered to be low risk for CDI induction (Spigaglia, 2016). However, tetR can be found in up to 41% of clinical *C. difficile* isolates and may be clinically significant since they represent reservoirs for genes encoding efflux and ribosomal protective proteins (Spigaglia, 2016). In this study, 69% of porcine strains presented a tetR phenotype, all of which carried the *tetM* gene on a conjugative transposon very similar to Tn*5397*. This element is the primary *tetM* encoding conjugative transposon found in *C. difficile* and like Tn*6194* is capable of intra- and inter-species transfer *in vitro* (Roberts and Mullany, 2011). The group II intron interrupting orf14 is a defining characteristic for Tn*5397* which was the first element of its kind to be found in a Gram-positive organism (Mullany et al., 1996; Spigaglia, 2016). Genetic studies show that in Tn*916* (a close relative of Tn*5397*), orf14 encodes a putative protein homologous in the C-terminal region to the invasion-associated protein p60 from *Listeria monocytogenes* (Köhler et al., 1991). Moreover, the p60 homolog of *Tn916* is essential for intercellular transposition, providing indirect evidence that the intron in Tn*5397*, which contains reverse transcriptase, RNA-binding, RNA splicing and zinc finger-like domains, undergoes splicing *in vivo* (Clewell et al., 1995; Roberts et al., 2001). The p60 homolog present in the Tn*5397* variant belongs to a large superfamily of N1pC/P60 peptidoglycan hydrolytic enzymes and is present within many Firmicutes including *Enterococcus* and *Clostridium*. To our knowledge, the variant Tn*5397* described in this study is novel and further demonstrates the heterogeneity seen among the Tn*916* family of transposons (Roberts and Mullany, 2011). The absence of the intron in the Tn*5397*-like element is unlikely to affect the element's ability to conjugate; however, further studies will be necessary to verify this.

*tetW* also encodes a ribosomal protective protein and can be found in a wide range of environmental and clinical bacteria (Spigaglia et al., 2008). The *tetW* element we report is different to that previously described in *C. difficile* (Spigaglia et al., 2008) but identical to the *tetW* gene from Tn*B1230* in *Butyrivibrio fibrisolvens*. This obligate anaerobic species is a predominant rumen commensal and capable of genetic exchange (*ermB*) with *C. difficile in vitro* (Robinson et al., 1981; Spigaglia et al., 2005). The absence of an upstream promoter necessary for tetracycline resistance may explain why those porcine strains harboring *tetW* but not *tetM* (P10 and P11) failed to show resistance *in vitro* (Spigaglia et al., 2005). This study also provides the first report in *C. difficile* of *tetA(P)* and *tetB(P)*, elements encoding efflux and ribosomal protective proteins, respectively. Further studies will be necessary to verify if the absence of genetic architecture provided by pCW3, the plasmid that normally harbors these elements in *C. perfringens*, is the reason strains harboring only *tetA(P)*/*tetB(P)* did not show resistance *in vitro*.

The acquisition and genomic integration of bacteriophages represent a major source of genetic diversity in *C. difficile* (Shan et al., 2012; Hargreaves and Clokie, 2014). The RT014 population in this study harbored numerous complete prophages belonging the *Caudovirales*, the order of tailed bacteriophages. All detected prophages contained a GC content not dissimilar to that of the *C. difficile* genome (28–30%) and putative integrase genes suggesting they have access to the lysogenic lifestyle. Several of the *C. difficile* phages identified in this study have been extensively studied *in vitro* revealing putative roles in the fitness and virulence of the host species. Studies show ΦC2, which was common to almost all RT014 genomes in this study is capable of mediating the transduction of Tn*6215*-encoded *ermB* resistance between laboratory strains of *C. difficile* (Goh et al., 2013). Siphovirus ΦCD38-2 and myovirus ΦCD27 have been shown to modulate toxin production in *C. difficile in vitro*, however, the genetic basis of the interaction is not yet understood (Roberts et al., 2014). Viral DNA identical to that of the *Clostridium* myovirus ΦMMP02 has been recovered from stool samples obtained from patients with CDI, indicating these phages are induced during infection (Roberts et al., 2014). Lastly, myovirus ΦCDHM1 has been found to contain *agr* gene homologs and therefore has the potential during phage lysogeny to influence expression, by a quorum signaling mechanism, of multiple genes associated with flagella assembly and toxin synthesis (Hargreaves et al., 2014).

Pigs are well-known amplification reservoirs for *C. difficile* and other enteric pathogens (Malik et al., 2011; Squire and Riley, 2013). Our data further confirms pigs are reservoirs for clinically important antimicrobial resistance elements, many of which are capable of reciprocal genetic exchange across large phylogenetic distances. Such promiscuous behavior provides *C. difficile* with a potential selective advantage over taxa inhabiting the same gut ecosystem, be it the pig or human intestinal tract.

The marked differences in antimicrobial resistance between the human and animal RT014 populations suggest limited genetic overlap and an absence of a common source, a finding which contrasts with the results of our evolutionary and phylogenetic analyses. However, it is important to note that the genomic elements mediating antimicrobial resistance as well as prophages are discrete parts of the highly dynamic accessory genome and their acquisition and loss from *C. difficile* occur under forces of selection such as antimicrobial exposure. Thus, the observed discordance may reflect different selective pressures in their most recent host environment (e.g., livestock vs. hospital and community settings). Use of tetracyclines and macrolides in animal husbandry is widespread, particularly for disease treatment and prevention (metaphylaxis; Jordan et al., 2009; Van Boeckel et al., 2015). Such use creates a massive selective pressure and an ideal environment for the development and spread of antibiotic resistance (Robinson et al., 2016). Conversely, the use of these agents in human medicine is relatively low, with neither antimicrobial ranking among the 10 most commonly prescribed antimicrobial agents in Australian hospitals (ACSQHC, 2015), however, they remain popular in the community. It is conceivable that within the healthcare system, the RT014 accessory genome is changing *in vivo* in response to a reduction in antimicrobial selective pressure. Furthermore, discordant phenotypes between bacterial clones are not without precedent. In a 2011 UK study, WGS was able to identify an *S. aureus* transmission event between patients during an MRSA outbreak, a connection which was initially refuted due to discordant tetracycline (*tetK*^+/−^) and penicillin (*BlaZ*^+/−^) genotypes and phenotypes (Eyre et al., 2012).

### Strains of RT014 from humans and pigs show similar virulence potential

The finding of similar and in some cases identical virulence loci in human and porcine strains of RT014 indicates a very similar virulence potential. Most significantly, all RT014 strains irrespective of host species or ST, harbored genes encoding large clostridial glucosylating toxins TcdA and TcdB, both major *C. difficile* virulence factors important for disease (Kuehne et al., 2010). Consistent with other clade 1 RTs, all RT014 genomes were negative for mutations in *tcdC*, a putative negative regulator of toxin production, and the genes encoding binary toxin (*cdtA*/*cdtB*) were both present as non-functional pseudogenes, having accumulated numerous frameshift mutations and in-frame stop codons (Curry et al., 2007; Gerding et al., 2014).

Additionally, we found all human and animal RT014 strains harbored similar, and sometimes identical alleles for virulence loci associated with motility (*fliC, fliD*), adhesion (*groEL* and *fbpA*), sporulation (*spoA*) as well as type IV pilin genes (Hennequin et al., 2001; Barketi-Klai et al., 2011; Pettit et al., 2014; Piepenbrink et al., 2015; Stevenson et al., 2015).

Another notable finding was presence in all RT014 genomes of an uncommon accessory gene regulator (*agr*) locus, *agr3*, the first such report in this lineage. Via a complex quorum-sensing system, the *agr* locus can both bolster and subvert *C. difficile* toxin synthesis and sporulation (Hargreaves et al., 2014). Locus type varies between different *C. difficile* lineages, for example, epidemic RTs 027 and 017 harbor an *agr2* locus (*agrBDAC* genes), whilst strains of RT012 harbor an *agr1* locus (*agrBD* only). Moreover, using isogenic mutants, *agr1* has been shown to be essential for pathogenesis in *C. difficile* (Darkoh et al., 2016). The *agr3* locus comprises syntenic *agrC, agrB* and *agrD* genes and has been found in strains of RTs 078 and 027 but also notably within the genome of both the prophage and natural lysogen of *C. difficile* ΦCDHM1, a finding which suggests horizontal gene transfer and a potentially novel way for phages to manipulate host behavior (Hargreaves et al., 2014). Further studies are ongoing to elucidate if the *agr3* locus is present exclusively within the RT014 host chromosome or lies within with any of the numerous prophage sequences found in this lineage.

### Sequence divergence in genes associated with pathogenicity and host-pathogen interaction largely correlate with ST affiliation

Sequence deviations in numerous conserved genes correlate with RT affiliation, a finding which further extends the concept of clonal *C. difficile* lineages (Dingle et al., 2011; Kurka et al., 2014). Taking this approach a step further, we analyzed sequence divergence in the same 14 genes from the study of Kurka et al. (2014) but also included a further 31 genes associated with pathogenicity, mobility, sporulation, antimicrobial resistance, and host-pathogen interaction. In our analysis, over two-thirds of the gene set showed 100% sequence conservation irrespective of host species or ST lineage, but differed from homologs in closely related clade 1 reference CD630. The majority of conserved loci encoded proteins involved in the essential host functions such as motility, sporulation, and protein synthesis but also antimicrobial resistance. These data complement the results of Kurka et al. indicating evolution in these genes is strongly associated with RT affiliation.

Conversely, we did find that genetic variability in a number of genes (and their proteins) correlated with two or three of the RT014 sublineages STs 2, 13, and 49. Variation was most notable in genes involved in pathogenicity (PaLoc) and host interaction (S-layer cassette) both regions of the *C. difficile* chromosome that have been shown to translocate by recombination and play an important role in clade evolution (Dingle et al., 2013, 2014). The RT014 S-layer cassette was particularly variable with four *slpA* allele types identified and was evolving under purifying selection. Forming an important interface between the bacterium and its host, the *C. difficile* S-layer evolves in response host immunological selection and plays a central role in adaption to life in the gastrointestinal tract. It is possible that sequence variability seen in this locus reflects time spent within different host species.

### The *C. difficile* RT014 lineage is characterized by a large diverse pan-genome and low levels of genome conservation

*C. difficile* is one of the most versatile bacterial pathogens and a model sympatric species. It possesses a large complex genome which diversifies through genetic exchange with a vast community of prokarya and archea present in both its primary habitat, the mammalian gastrointestinal tract, and a wide range of secondary habitats including soil, water, and non-mammalian species (Knight et al., 2015a). Here, we show *C. difficile* RT014 is characterized by a large pan-genome of 7587 genes, itself comprising a core of 2296 genes (representing 30.3% of the pan-genome) and an accessory gene repertoire totalling 5291 genes. Previous studies have yielded varying estimates of the *C. difficile* core genome ranging from ~600 to 4100 genes, comprising anywhere between 16 and 40% of the *C. difficile* genomes under analysis (Janvilisri et al., 2009; Scaria et al., 2010; Treangen et al., 2014). Such variation in size is due to inherent differences in (i) methodology (some used microarray and others used different gene prediction and ortholog clustering algorithms), (ii) the use of strict vs relaxed core genome definitions, and (iii) sample size/diversity (some were limited to only a few isolates or an individual strain lineage). Nonetheless, taken together these studies show *C. difficile* displays ultra-low levels of genome conservation, a trait rarely seen in bacteria and lower than other bacterial species considered to have high levels of genetic variability such as *Campylobacter jejuni* (59.2%), *Helicobacter pylori* (58.5%), *Streptococcus pneumoniae* (46.5%), and *E. coli* (~40.0%; Welch et al., 2002; Hiller et al., 2007; Lu et al., 2013; Vernikos et al., 2015).

The open or closed nature of a bacterial pan-genome is dependent on a number of factors including the host species' capacity to acquire and replicate exogenous DNA, the relative rate of evolution, and diversification and lifestyle of the species (Tettelin et al., 2008). Our analysis shows the RT014 pan-genome is open and characterized by significant variability and plasticity, a remarkable finding considering the relatively small number of strains analyzed, and one which will likely only increase as more RT014 genomes are sequenced. The large accessory genome and presence of a large number of singletons (41% of the accessory genome) suggests the RT014 genome is highly submissive to lateral transfer of exogenous DNA, a trait emphasized by the diverse collection of transposons and phages (intact and incomplete) identified in this study.

The respective pan-genomes (and proteomes) of the porcine and human RT014 strain populations were very similar. Taken together with our microevolutionary analysis, this suggests RT014 has the capability and propensity to move freely between porcine and human populations. This lineage appears well-adapted to multiple animal hosts having been recovered from numerous diseased and colonized species including cattle, horses, cats, dogs, hares, rabbits, goats, racoons and multiple avian species (Janezic et al., 2014). By occupying niches within multiple host species, the RT014 lineage is able to access and exchange DNA with an enormously diverse metagenome, therefore greatly enhancing its ability to adapt to fluctuating environmental factors and its likelihood of success.

## Concluding remarks

In conclusion, the present study provides novel insights on the genetic variability and strain relatedness of RT014, a *C. difficile* lineage of emerging One Health importance. We show for the first time that human and porcine strains of RT014 do not form distinct populations; rather strains share a recent evolutionary history with evidence of long-range inter-species transmission. Moreover, we show that the RT014 lineage is characterized by a large open pan-genome, the presence of numerous prophages and clinically important antimicrobial resistance elements.

Throughout Australia, gross contamination of the piggery environment with *C. difficile* spores and agricultural recycling of piggery effluent are now commonplace and undoubtedly result in spillover contamination of vegetables grown in organically enriched soil and compost in the community setting. Reducing the levels of *C. difficile* spores in the piggery environment is of paramount importance, not only for mitigating the risk of community acquisition but also for improving animal health. In hospitals, *C. difficile* spore transmission and overall CDI rates can be significantly reduced through stringent infection control measures such as case isolation, reduced use of third-generation cephalosporins and fluoroquinolones, hand hygiene and deep environmental cleaning (Thomas et al., 2002; Price et al., 2010). In pig populations, the sheer scale of the potential *C. difficile* reservoir combined with the extreme resilience and high transmissibility of *C. difficile* spores and relatively unfettered use of antibiotics means infection control measures cannot be easily implemented and maintained. This is further complicated in Australia by a lack of acceptance of *C. difficile* as a pathogen in animal populations (despite abundant evidence to the contrary) and at times disagreement between clinicians, veterinarians and the livestock industry regarding appropriate risk management of *C. difficile* in animal populations (Riley, 2009; Squire and Riley, 2013).

Finally, CDI is a complex phenomenon and our understanding of CDI transmission dynamics, particularly in the food chain and community setting is still in its infancy. Ongoing molecular and phenotypic surveillance of *C. difficile* strains in humans, animals, food, and the environment is imperative if we are to identify opportunities for interventions and reduce the overall CDI burden. As we have seen with RT014, WGS will surely play a central role in this, providing a level of discrimination far beyond that achievable by conventional typing methodologies.

## Author contributions

DK designed and performed all experimental and bioinformatics work, analyzed all the data, and co-wrote the manuscript. MS and TR designed the study, analyzed the data, and co-wrote the manuscript. DC critically revised the manuscript and assisted with data analysis. All authors have read and approved the final manuscript.

## Funding

This study was partially supported by a grant from the Australian Research Council (DP150104670). DK is funded by an Australian Postgraduate Award conferred by The University of Western Australia.

### Conflict of interest statement

The authors declare that the research was conducted in the absence of any commercial or financial relationships that could be construed as a potential conflict of interest.
