# Supplementary material for: Genome Analysis of Clostridium difficile PCR Ribotype 014 Lineage in Australian Pigs and Humans Reveals a Diverse Genetic Repertoire and Signatures of Long-Range Interspecies Transmission
Source: Front Microbiol. 2017 Jan 11;7:2138. doi: 10.3389/fmicb.2016.02138 (PMC5225093; doi:10.3389/fmicb.2016.02138)
Supplement: Supplementary file 1 [file Table1.pdf]

## *Supplementary Material*

**Knight DR*, Squire MM, Collins DA and Riley TV** (2016). Genome analysis of *Clostridium difficile* PCR ribotype 014 lineage in Australian pigs and humans reveals a diverse genetic repertoire and signatures of long-range interspecies transmission. *Front. Microbiol.* **7**:2138.
doi: 10.3389/fmicb.2016.02138

***Correspondence:** Daniel R. Knight; daniel.knight@uwa.edu.au

**Supplementary Table 1 | Metrics for *C. difficile* RT014 genomes, n=44**

| ID | Genome size (bp) | GC% | N genes | N CDS | CDS Length | Min CDS length | Max CDS length | N tRNA | N rRNA | N CRISPR | Total CDS Length (Mbp) | Coding % | Coding density (CDS/Mbp) | N Contigs | Largest Contig (bp) | N50 |
|---|---|---|---|---|---|---|---|---|---|---|---|---|---|---|---|---|
| P1 | 4306274 | 28.73 | 4119 | 4034 | 753 | 74 | 7101 | 70 | 14 | 12 | 3.60 | 83.5% | 937 | 142 | 679621 | 107246 |
| P2 | 4357743 | 28.62 | 4074 | 4004 | 768 | 74 | 7101 | 58 | 11 | 12 | 3.61 | 82.9% | 919 | 100 | 785945 | 111724 |
| P3 | 4375492 | 28.75 | 4106 | 4020 | 759 | 63 | 7101 | 70 | 15 | 12 | 3.60 | 82.2% | 919 | 196 | 414802 | 75262 |
| P4 | 4393795 | 28.75 | 4069 | 3986 | 756 | 74 | 7101 | 67 | 15 | 11 | 3.54 | 80.7% | 907 | 184 | 421420 | 68581 |
| P5 | 4366415 | 29.12 | 4305 | 4248 | 744 | 74 | 8133 | 42 | 14 | 11 | 3.75 | 85.8% | 973 | 289 | 940433 | 38916 |
| P6 | 4175123 | 28.63 | 3792 | 3748 | 786 | 74 | 8133 | 32 | 11 | 13 | 3.44 | 82.4% | 898 | 152 | 289992 | 73148 |
| P7 | 4293431 | 29.19 | 4261 | 4204 | 741 | 74 | 8133 | 41 | 15 | 10 | 3.68 | 85.6% | 979 | 360 | 679500 | 33918 |
| P8 | 4216883 | 29.46 | 4175 | 4120 | 690 | 74 | 8133 | 41 | 13 | 9 | 3.59 | 85.0% | 977 | 401 | 730980 | 19469 |
| P9 | 4397662 | 29.28 | 4127 | 4070 | 717 | 74 | 7680 | 44 | 12 | 10 | 3.57 | 81.1% | 925 | 390 | 748992 | 32136 |
| P10 | 4245597 | 28.88 | 3870 | 3793 | 768 | 74 | 7959 | 62 | 14 | 11 | 3.45 | 81.3% | 893 | 321 | 526705 | 31341 |
| P11 | 4324910 | 29.15 | 3906 | 3840 | 738 | 74 | 8133 | 51 | 14 | 11 | 3.43 | 79.4% | 888 | 422 | 540246 | 26246 |
| P12 | 4303866 | 28.39 | 4044 | 3967 | 768 | 74 | 7101 | 63 | 13 | 11 | 3.58 | 83.2% | 922 | 64 | 1036193 | 201820 |
| P13 | 4242084 | 28.38 | 3932 | 3883 | 777 | 74 | 7101 | 41 | 7 | 11 | 3.53 | 83.3% | 915 | 61 | 1399922 | 234188 |
| P14 | 4246188 | 28.41 | 3913 | 3841 | 774 | 67 | 7101 | 59 | 12 | 10 | 3.50 | 82.3% | 905 | 103 | 851845 | 112932 |
| P15 | 4316672 | 28.53 | 4017 | 3963 | 771 | 74 | 7101 | 42 | 11 | 12 | 3.56 | 82.6% | 918 | 101 | 1429538 | 135018 |
| P16 | 4318597 | 28.65 | 4270 | 4207 | 702 | 74 | 8007 | 49 | 13 | 19 | 3.49 | 80.9% | 974 | 259 | 233681 | 41795 |
| H1 | 4260101 | 28.64 | 3916 | 3822 | 777 | 71 | 7101 | 77 | 16 | 11 | 3.49 | 81.9% | 897 | 110 | 688013 | 126300 |

| ID | Genome size (bp) | GC% | N genes | N CDS | CDS Length | Min CDS length | Max CDS length | N tRNA | N rRNA | N CRISPR | Total CDS Length (Mbp) | Coding % | Coding density (CDS/Mbp) | N Contigs | Largest Contig (bp) | N50 |
|---|---|---|---|---|---|---|---|---|---|---|---|---|---|---|---|---|
| H2 | 4343405 | 28.48 | 4074 | 4018 | 768 | 74 | 7101 | 43 | 12 | 10 | 3.62 | 83.4% | 925 | 83 | 310868 | 139344 |
| H3 | 4159157 | 28.54 | 3834 | 3756 | 783 | 74 | 7101 | 65 | 12 | 13 | 3.44 | 82.7% | 903 | 90 | 745387 | 184247 |
| H4 | 4171144 | 28.43 | 3825 | 3752 | 792 | 74 | 7101 | 59 | 13 | 10 | 3.48 | 83.3% | 900 | 59 | 1317818 | 202408 |
| H5 | 4111116 | 28.5 | 3754 | 3671 | 786 | 72 | 7101 | 71 | 11 | 10 | 3.39 | 82.4% | 893 | 92 | 517042 | 139312 |
| H6 | 4213817 | 28.42 | 3846 | 3792 | 789 | 74 | 7101 | 44 | 9 | 11 | 3.49 | 82.8% | 900 | 70 | 782378 | 229392 |
| H7 | 4140387 | 28.43 | 3769 | 3705 | 792 | 74 | 7101 | 56 | 7 | 8 | 3.44 | 83.0% | 895 | 65 | 651455 | 164735 |
| H8 | 4276164 | 28.44 | 3976 | 3901 | 765 | 74 | 7101 | 66 | 8 | 11 | 3.51 | 82.2% | 912 | 119 | 455052 | 98541 |
| H9 | 4023709 | 28.33 | 3883 | 3821 | 783 | 74 | 7101 | 53 | 8 | 8 | 3.51 | 87.2% | 950 | 86 | 320737 | 109457 |
| H10 | 4321348 | 29.29 | 3976 | 3916 | 773 | 74 | 8007 | 46 | 13 | 17 | 3.55 | 82.2% | 906 | 64 | 601448 | 209929 |
| H11 | 4188619 | 28.56 | 3845 | 3766 | 777 | 74 | 7101 | 65 | 13 | 13 | 3.43 | 81.9% | 899 | 127 | 356540 | 84471 |
| H12 | 4092688 | 28.49 | 3888 | 3824 | 750 | 74 | 7101 | 51 | 12 | 12 | 3.36 | 82.2% | 934 | 120 | 242455 | 71487 |
| H13 | 4240966 | 28.49 | 3896 | 3815 | 780 | 74 | 7101 | 65 | 15 | 11 | 3.49 | 82.4% | 900 | 98 | 547759 | 107981 |
| H14 | 4261108 | 28.74 | 3872 | 3799 | 774 | 66 | 7101 | 61 | 11 | 12 | 3.45 | 81.0% | 892 | 174 | 529871 | 77959 |
| H15 | 4159033 | 28.53 | 3820 | 3751 | 786 | 74 | 7101 | 57 | 11 | 13 | 3.44 | 82.8% | 902 | 85 | 1519890 | 199533 |
| H16 | 4181626 | 28.76 | 3782 | 3722 | 768 | 74 | 8133 | 41 | 18 | 11 | 3.42 | 81.7% | 890 | 168 | 326021 | 73022 |
| H17 | 4175123 | 28.63 | 4047 | 3972 | 702 | 68 | 8133 | 52 | 22 | 13 | 3.55 | 85.0% | 951 | 152 | 297923 | 73148 |
| H18 | 4201620 | 28.86 | 3806 | 3745 | 762 | 71 | 8133 | 41 | 19 | 13 | 3.44 | 81.9% | 891 | 184 | 487487 | 51608 |
| H19 | 4318953 | 28.95 | 4032 | 3965 | 753 | 74 | 8133 | 53 | 13 | 12 | 3.49 | 80.9% | 918 | 350 | 452553 | 27075 |
| H20 | 4304209 | 29.25 | 3885 | 3809 | 723 | 71 | 8877 | 53 | 22 | 13 | 3.45 | 80.2% | 885 | 254 | 339779 | 45013 |
| H21 | 4284079 | 28.95 | 3877 | 3785 | 762 | 72 | 8133 | 66 | 25 | 10 | 3.52 | 82.1% | 884 | 112 | 444046 | 125344 |
| H22 | 4106423 | 28.69 | 3733 | 3663 | 780 | 74 | 7101 | 57 | 12 | 10 | 3.33 | 81.0% | 892 | 398 | 296973 | 21492 |
| H23 | 4259443 | 29.3 | 3800 | 3731 | 738 | 72 | 8133 | 42 | 26 | 11 | 3.43 | 80.5% | 876 | 164 | 671044 | 101677 |
| H24 | 4205942 | 29.12 | 3728 | 3654 | 765 | 72 | 8133 | 49 | 24 | 10 | 3.44 | 81.7% | 869 | 152 | 776075 | 135789 |
| Ox1533 | 4266433 | 28.26 | 3933 | 3866 | 783 | 74 | 8577 | 53 | 13 | 12 | 3.53 | 82.7% | 906 | 82 | 464892 | 104490 |
| Ox593 | 4257373 | 28.5 | 3980 | 3914 | 770 | 74 | 7101 | 53 | 12 | 12 | 3.53 | 82.8% | 919 | 59 | 906054 | 19178 |
| Ox1475 | 4302598 | 28.77 | 4001 | 3933 | 780 | 74 | 7101 | 53 | 14 | 11 | 3.59 | 83.3% | 914 | 385 | 492254 | 18427 |
| ATCC 43600 | 4202469 | 28.58 | 3863 | 3816 | 789 | 74 | 8007 | 32 | 14 | 14 | 3.50 | 83.2% | 908 | 27 | 1854258 | 564956 |
