# Supplementary material for: Genome Analysis of Clostridium difficile PCR Ribotype 014 Lineage in Australian Pigs and Humans Reveals a Diverse Genetic Repertoire and Signatures of Long-Range Interspecies Transmission
Source: Front Microbiol. 2017 Jan 11;7:2138. doi: 10.3389/fmicb.2016.02138 (PMC5225093; doi:10.3389/fmicb.2016.02138)
Supplement: Supplementary file 2 [file Table2.pdf]

Supplementary Material

Knight DR*, Squire MM, Collins DA and Riley TV (2016). Genome analysis of *Clostridium difficile* PCR ribotype 014 lineage in Australian pigs and humans reveals a diverse genetic repertoire and signatures of long-range interspecies transmission. *Front. Microbiol.* **7**:2138.
doi: 10.3389/fmicb.2016.02138

*Correspondence: Daniel R. Knight; daniel.knight@uwa.edu.au

Supplementary Table 2 | Summary of complete prophages detected in *C. difficile* RT014 genomes, n=44

Genome	Origin	ST	Most Common Prophage	Score†	Length (Kb)	N ORFs	GC%
P1	Porcine	13	Clostr_ΦC2_NC_009231	150	40.8	83	32.11
P1	Porcine	13	Clostr_ΦMMP02_NC_019421	150	57.3	90	29.85
P2	Porcine	13	Clostr_ΦC2_NC_009231	100	16.6	29	30.13
P3	Porcine	13	Clostr_ΦC2_NC_009231	150	105.7	143	27.77
P3	Porcine	13	Clostr_ΦC2_NC_009231	100	21.4	32	34.20
P3	Porcine	13	Clostr_ΦMMP02_NC_019421	150	72.7	114	30.35
P5	Porcine	49	Clostr_CDMH1_NC_024144	150	80.0	101	28.74
P5	Porcine	49	Clostr_ΦC2_NC_009231	130	50.5	70	30.06
P5	Porcine	49	Clostr_ΦCD6356_NC_015262	150	87.6	106	29.59
P5	Porcine	49	Clostr_ΦMMP02_NC_019421	100	27.4	29	31.67
P7	Porcine	49	Clostr_CDMH1_NC_024144	100	17.6	29	29.49
P7	Porcine	49	Clostr_ΦC2_NC_009231	150	51.7	79	28.83
P7	Porcine	49	Clostr_ΦC2_NC_009231	100	46.7	53	29.61
P7	Porcine	49	Clostr_ΦCD6356_NC_015262	150	59.7	80	29.82
P7	Porcine	49	Clostr_ΦMMP02_NC_019421	150	94.1	103	32.73
P8	Porcine	49	Clostr_ΦCD6356_NC_015262	150	59.9	74	31.33
P8	Porcine	49	Clostr_ΦMMP02_NC_019421	150	89.0	116	29.36
P9	Porcine	49	Clostr_CDMH1_NC_024144	110	31.1	37	29.06
P9	Porcine	49	Clostr_ΦCD27_NC_011398	100	32.1	37	29.99
P9	Porcine	49	Clostr_ΦCD6356_NC_015262	150	54.2	66	28.05
P9	Porcine	49	Clostr_ΦMMP02_NC_019421	100	70.6	87	27.08
P10	Porcine	13	Clostr_ΦC2_NC_009231	150	73.9	101	29.16
P11	Porcine	13	Clostr_ΦC2_NC_009231	110	30.1	56	31.61
P12	Porcine	13	Clostr_ΦC2_NC_009231	150	41.9	59	28.78
P12	Porcine	13	Clostr_ΦCD6356_NC_015262	150	67.5	110	29.60
P12	Porcine	13	Clostr_ΦMMP02_NC_019421	150	39.0	46	30.18
P13	Porcine	13	Clostr_ΦC2_NC_009231	150	41.9	59	28.78
P13	Porcine	13	Clostr_ΦCD6356_NC_015262	150	49.9	80	29.25
P13	Porcine	13	Clostr_ΦMMP02_NC_019421	150	47.4	68	29.42
P14	Porcine	13	Clostr_ΦC2_NC_009231	150	41.9	58	28.78
P14	Porcine	13	Clostr_ΦMMP02_NC_019421	150	64.8	77	30.32
P15	Porcine	13	Clostr_CDMH1_NC_024144	100	17.2	30	30.27
P15	Porcine	13	Clostr_ΦC2_NC_009231	130	34.0	51	30.20

Genome	Origin	ST	Most Common Prophage	Score[†]	Length (Kb)	N ORFs	GC%
P15	Porcine	13	Clostr_ΦC2_NC_009231	120	49.6	49	29.13
P15	Porcine	13	Clostr_ΦC2_NC_009231	110	24.8	43	29.55
P16	Porcine	2	Clostr_ΦC2_NC_009231	140	24.3	40	29.74
P16	Porcine	2	Clostr_ΦMMP02_NC_019421	110	53.0	76	30.35
H1	Human	13	Clostr_ΦC2_NC_009231	150	48.7	78	28.85
H2	Human	2	Clostr_ΦC2_NC_009231	150	56.7	89	28.57
H2	Human	2	Clostr_ΦCD27_NC_011398	150	32.9	57	28.77
H2	Human	2	Clostr_ΦCD6356_NC_015262	150	96.6	108	28.75
H3	Human	2	Clostr_CDMH1_NC_024144	100	12.2	18	29.33
H3	Human	2	Clostr_ΦC2_NC_009231	130	39.4	63	31.16
H5	Human	2	Clostr_ΦC2_NC_009231	150	72.5	95	28.39
H6	Human	13	Clostr_ΦC2_NC_009231	150	56.5	88	28.53
H8	Human	49	Clostr_CDMH1_NC_024144	120	28.6	30	28.89
H8	Human	49	Clostr_ΦC2_NC_009231	130	63.8	73	29.81
H8	Human	49	Clostr_ΦC2_NC_009231	150	103.7	125	30.37
H9	Human	2	Clostr_ΦMMP02_NC_019421	150	50.7	76	28.74
H9	Human	2	Clostr_ΦSM101_NC_008265	100	29.9	30	26.67
H10	Human	2	Clostr_ΦC2_NC_009231	100	34.8	61	28.09
H11	Human	2	Clostr_CDMH1_NC_024144	140	51.8	74	30.99
H11	Human	2	Clostr_ΦC2_NC_009231	150	67.8	108	31.26
H12	Human	2	Clostr_ΦC2_NC_009231	100	34.7	64	28.16
H13	Human	13	Clostr_ΦC2_NC_009231	150	48.7	77	28.85
H14	Human	2	Clostr_ΦCD27_NC_011398	110	51.6	77	29.04
H15	Human	2	Clostr_CDMH1_NC_024144	130	16.9	26	29.44
H19	Human	13	Clostr_ΦC2_NC_009231	120	52.2	77	37.37
H19	Human	13	Clostr_ΦC2_NC_009231	100	22.5	38	30.11
H19	Human	13	Clostr_ΦMMP02_NC_019421	140	84.1	85	32.24
H19	Human	13	Clostr_ΦMMP02_NC_019421	150	41.6	73	35.07
H21	Human	2	Clostr_ΦC2_NC_009231	150	56.8	86	28.55
H22	Human	2	Clostr_ΦC2_NC_009231	150	99.6	101	28.88
H23	Human	2	Clostr_ΦC2_NC_009231	150	64.6	95	28.23
H24	Human	2	Clostr_ΦC2_NC_009231	140	55.7	86	28.99
Ox1475	Human	2	Clostr_ΦC2_NC_009231	150	101.2	130	27.12
Ox1475	Human	2	Clostr_ΦC2_NC_009231	100	32.2	54	28.41
Ox1475	Human	2	Clostr_ΦCD38_2_NC_015568	120	55.0	61	31.34
Ox1533	Human	13	Clostr_ΦC2_NC_009231	140	44.0	73	28.12
Ox893	Human	49	Clostr_CDMH1_NC_024144	110	27.3	39	28.80
Ox893	Human	49	Clostr_ΦC2_NC_009231	150	108.2	157	29.97
Ox893	Human	49	Clostr_ΦMMP02_NC_019421	150	89.4	133	29.10
ATCC43600	Human	2	Clostr_ΦC2_NC_009231	120	16.9	23	29.60
CD630	Human	54	Clostr_ΦC2	140	55.8	88	28.45
CD630	Human	54	Clostr_ΦC2	140	57.3	68	29.45

CD630, RT012 reference strain (Genbank AM180355)
ORF, open reading frame
[†], prophages were scored on the principle of completeness (see Methods)
