# Supplementary material for: Genome Analysis of Clostridium difficile PCR Ribotype 014 Lineage in Australian Pigs and Humans Reveals a Diverse Genetic Repertoire and Signatures of Long-Range Interspecies Transmission
Source: Front Microbiol. 2017 Jan 11;7:2138. doi: 10.3389/fmicb.2016.02138 (PMC5225093; doi:10.3389/fmicb.2016.02138)
Supplement: Supplementary file 3 [file Image1.pdf]

Supplementary Material

Knight DR*, Squire MM, Collins DA and Riley TV (2016). Genome analysis of *Clostridium difficile* PCR ribotype 014 lineage in Australian pigs and humans reveals a diverse genetic repertoire and signatures of long-range interspecies transmission. *Front. Microbiol.* **7**:2138. doi: 10.3389/fmicb.2016.02138

*Correspondence: Daniel R. Knight; daniel.knight@uwa.edu.au

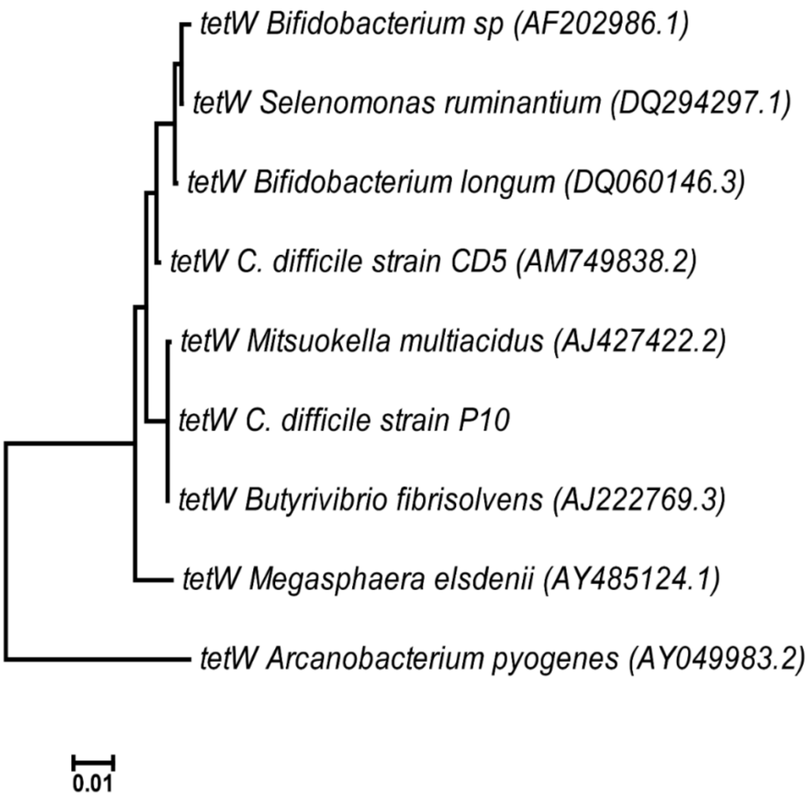

Supplementary Image 1 | *tetW* phylogeny

Mid-point rooted Neighbor-Joining tree generated from *tetW* nucleotide sequences (1920bp) from *C. difficile* strain P10 (representative of all *tetW* positive genomes, *n*=8) and seven homologous elements in related species.
