# Supplementary material for: Genome Analysis of Clostridium difficile PCR Ribotype 014 Lineage in Australian Pigs and Humans Reveals a Diverse Genetic Repertoire and Signatures of Long-Range Interspecies Transmission
Source: Front Microbiol. 2017 Jan 11;7:2138. doi: 10.3389/fmicb.2016.02138 (PMC5225093; doi:10.3389/fmicb.2016.02138)

Supplementary Material

Knight DR*, Squire MM, Collins DA and Riley TV (2016). Genome analysis of *Clostridium difficile* PCR ribotype 014 lineage in Australian pigs and humans reveals a diverse genetic repertoire and signatures of long-range interspecies transmission. *Front. Microbiol.* **7**:2138. doi: 10.3389/fmicb.2016.02138

*Correspondence: Daniel R. Knight; daniel.knight@uwa.edu.au

Supplementary Image 2 (A-N) | Sequence divergence in genes associated with virulence and host-pathogen interaction

Trees **A-N** depict Neighbor-Joining phylogenies for 14 genes found to display nucleotide sequence divergence across the 44 *C. difficile* RT014 strain collection. Gene tree legends: **A**, *atpA;* **B**, *rpoA;* **C**, *rpoB;* **D**, *rpoC;* **E**, *blaR;* **F**, *tcdR;* **G**, *tcdB;* **H**, *tcdC;* **I**, *slpA;* **J**, *cwp2;* **K**, *cwp11;* **L**, *cwp66;* **M**, *cwp25* and **N**, *agrB*. The ST lineage is represented by coloured circles; ST2 (●), ST13 (●) and ST49 (●). For comparative purposes, corresponding gene sequences from *C. difficile* reference genome CD630 (▲) are included in each phylogeny. The *agrB* gene sequence from CDR20291 (◆) is also included (tree **N**). For trees **G** and **H**, taxa labels for divergent *tcdB* and *tcdC* sequences are shown (H4, H9, H21 and H22). Similarly, for trees **I-L**, a taxa label for divergent *slpA*, *cwp66*, *cwp2* and *cwp11* sequences from human strain Ox1475 are shown. For tree **I** (*slpA*), the corresponding allele types are shown (Dingle *et al.*, 2013). Sequences were aligned using MUSCLE and Neighbor-Joining trees were generated in MEGA6 with evolutionary distances calculated using the Tajima-Nei model. Scale bar shows the number of substitutions per site. All trees are mid-point rooted, supported by 500 bootstrap replicates (not shown).

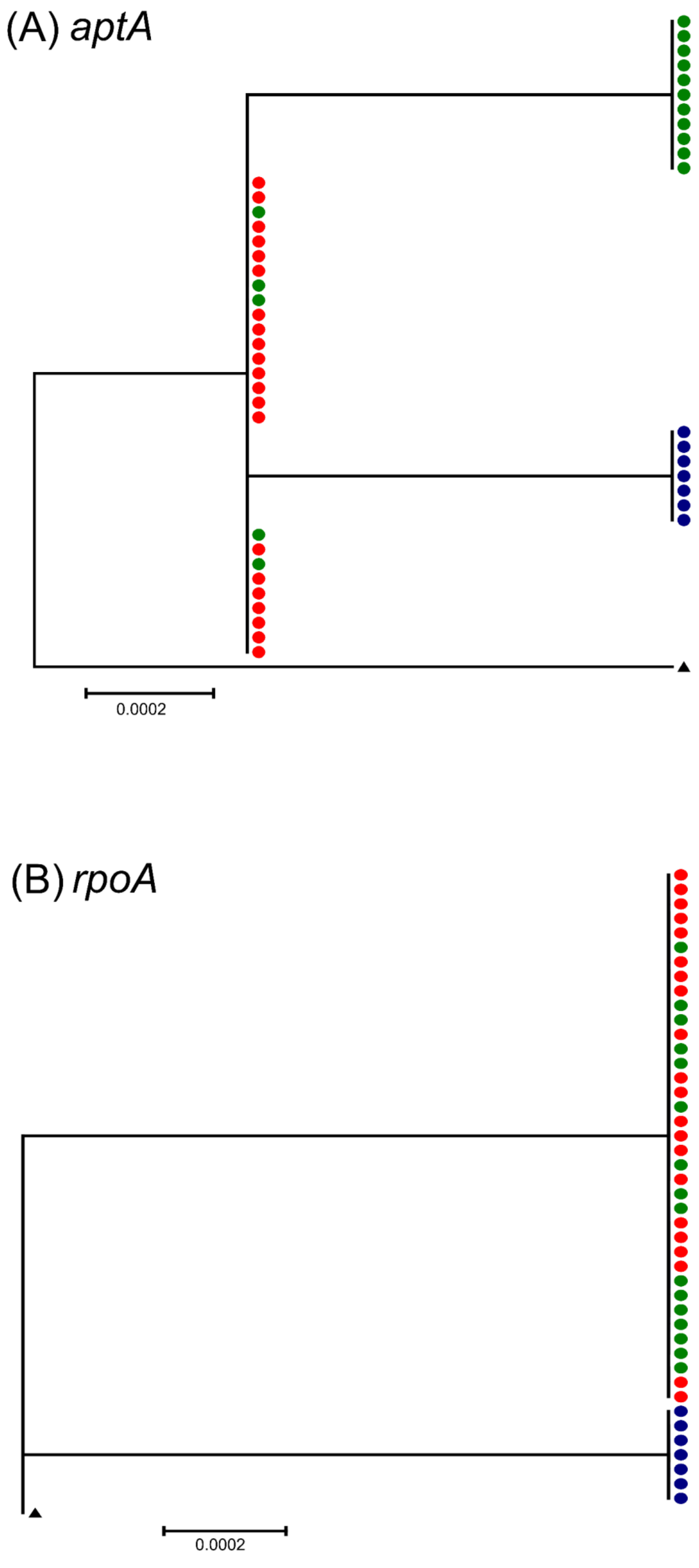

(A) aptA
0.0002
(B) rpoA
0.0002

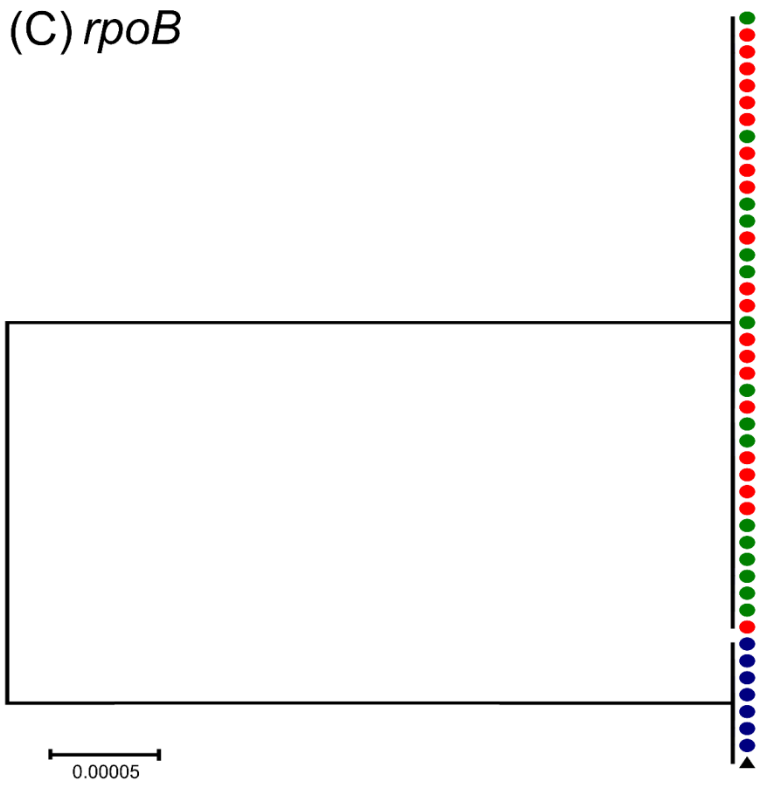
(C) rpoB
0.00005

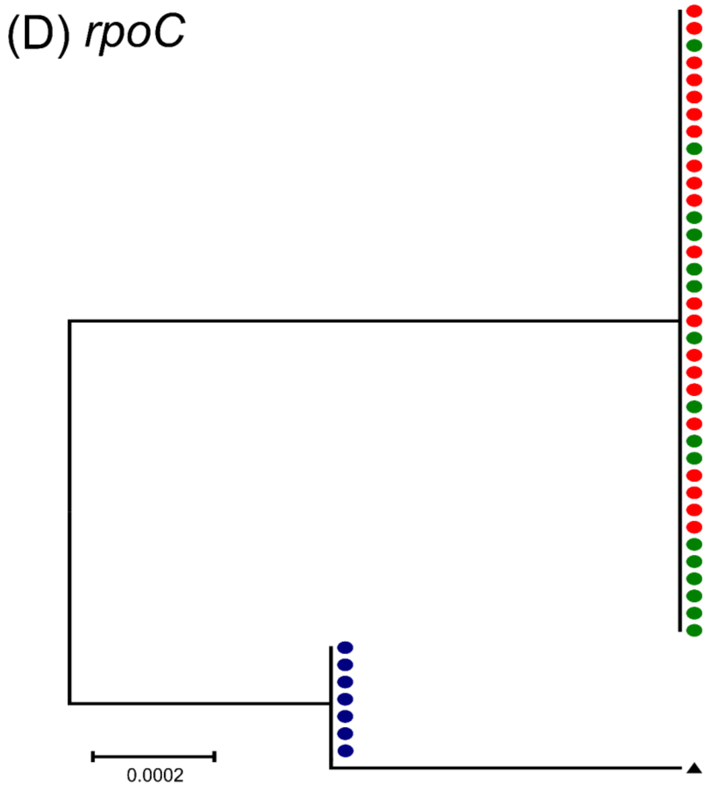
(D) rpoC
0.0002

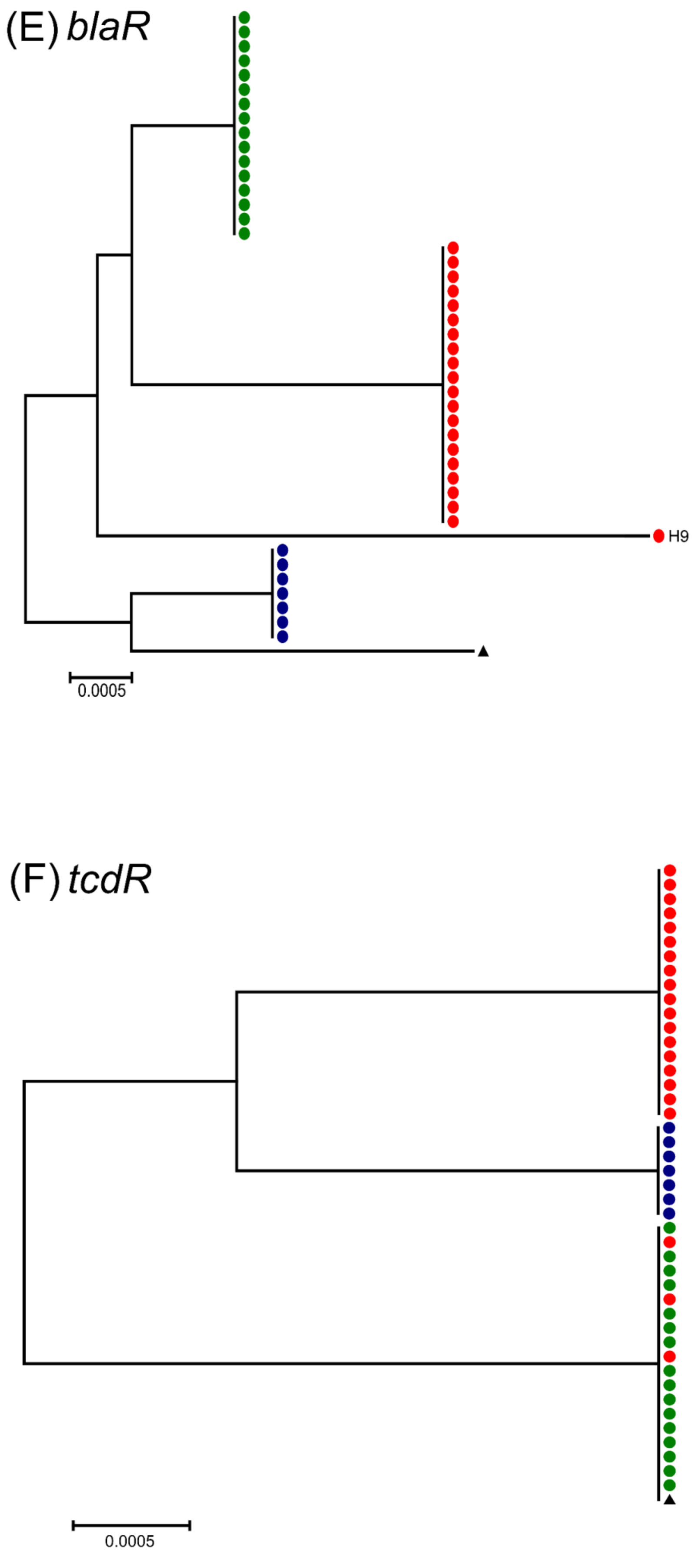

(E) blaR
H9
0.0005
(F) tcdR
0.0005

(G) *tcdB*

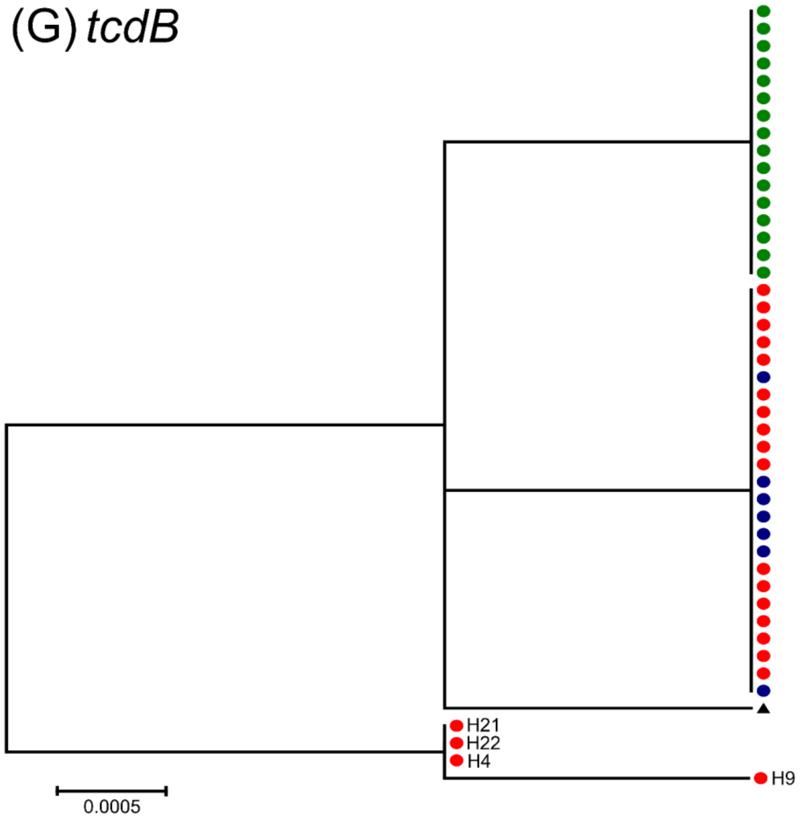

(H) *tcdC*

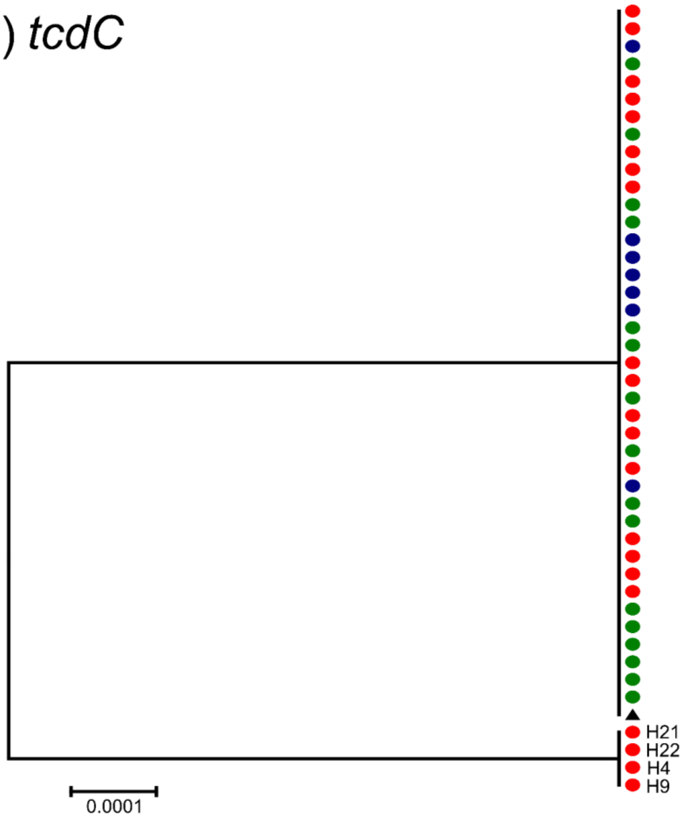

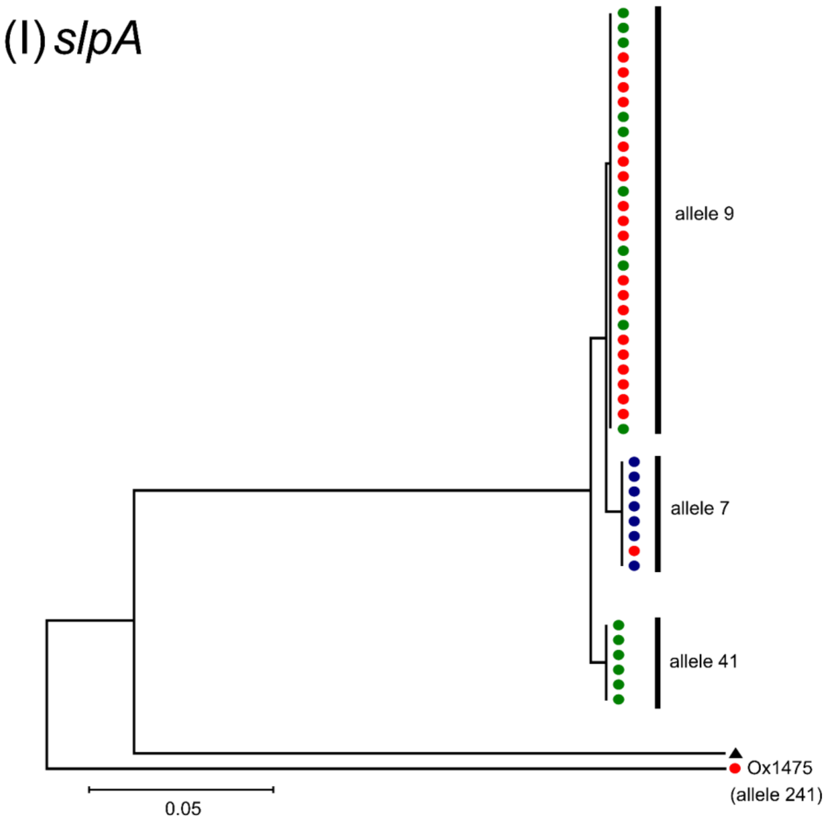

(I) slpA
allele 9
allele 7
allele 41
Ox1475
(allele 241)
0.05

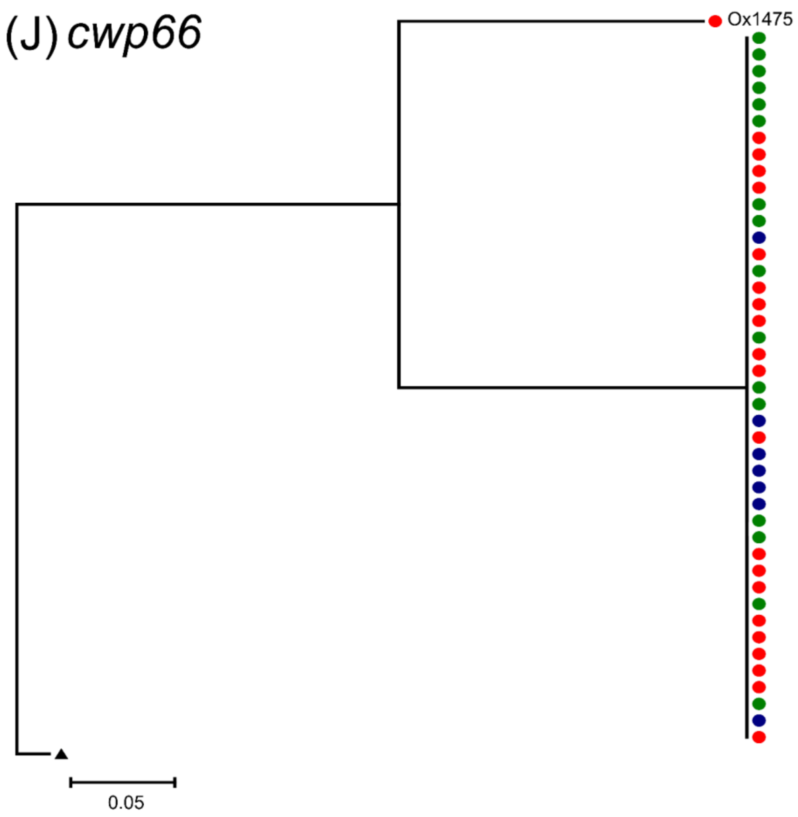

(J) cwp66
Ox1475
0.05

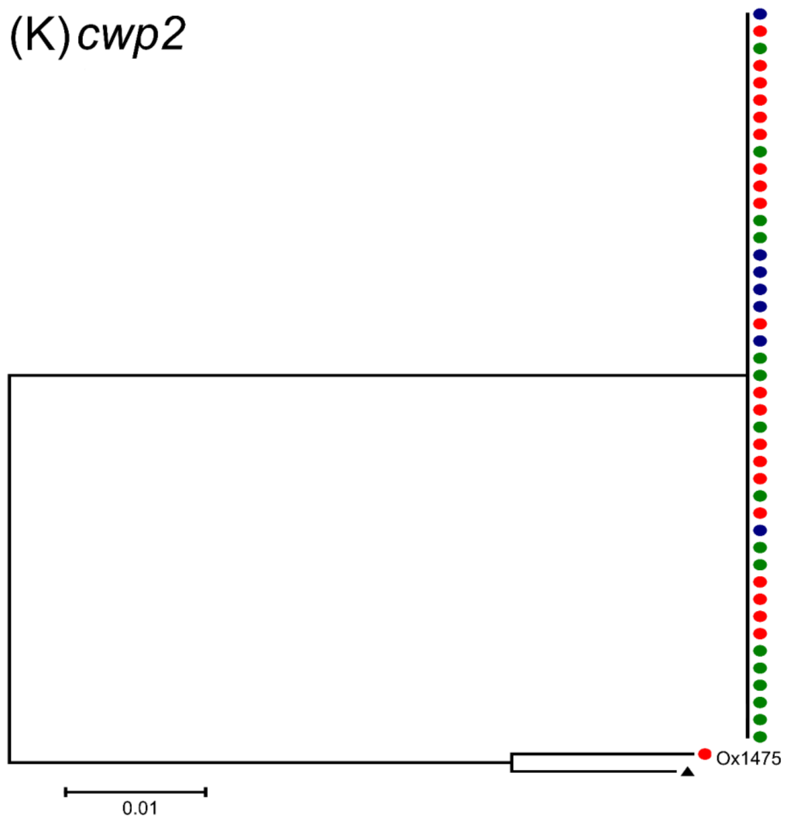
(K) *cwp2*
Ox1475
0.01

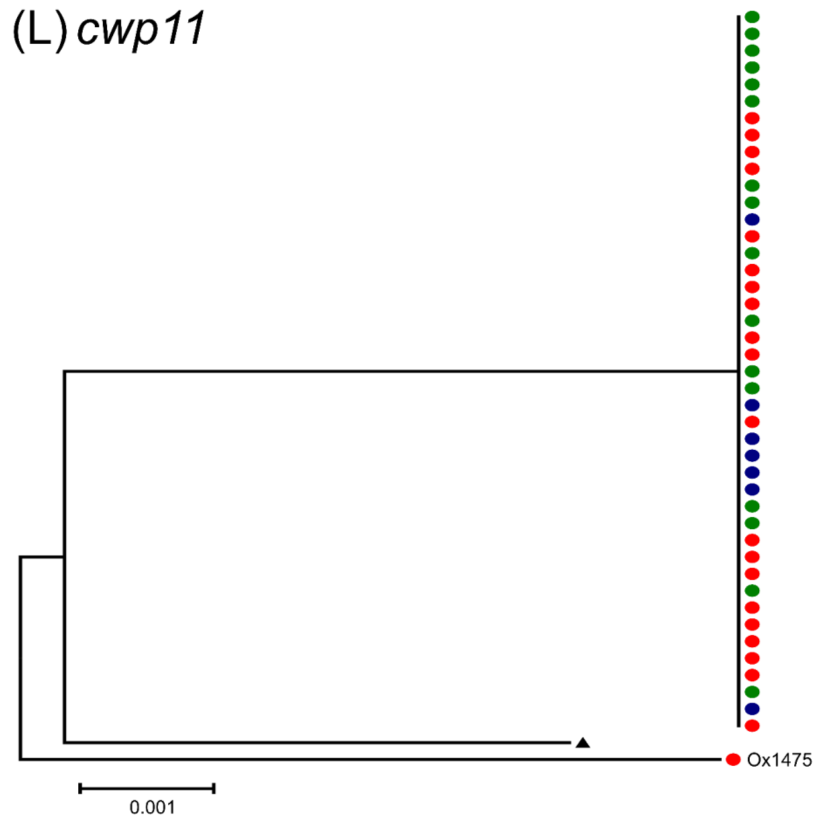
(L) *cwp11*
Ox1475
0.001

(M) *cwp25*

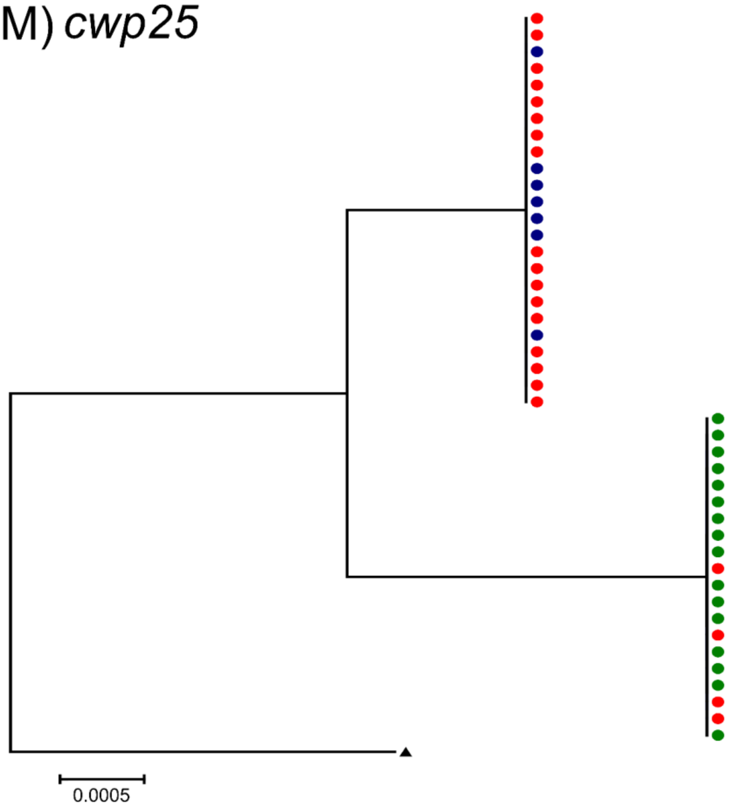

(N) *agrB*

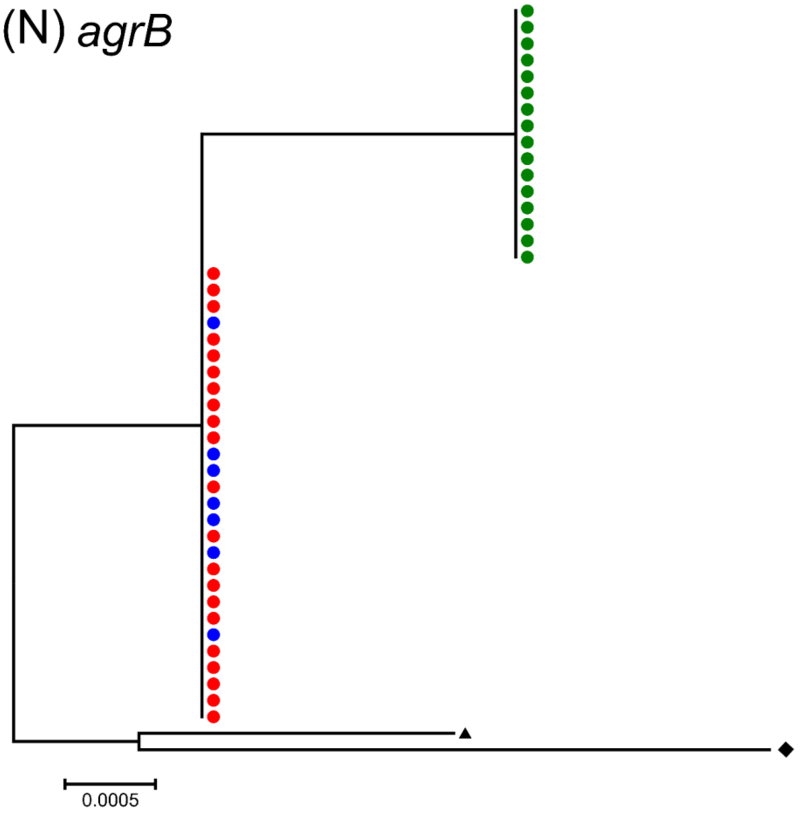

Supplement: Supplementary file 4 [file Image2.pdf]
