# Supplementary material for: Genome Analysis of Clostridium difficile PCR Ribotype 014 Lineage in Australian Pigs and Humans Reveals a Diverse Genetic Repertoire and Signatures of Long-Range Interspecies Transmission
Source: Front Microbiol. 2017 Jan 11;7:2138. doi: 10.3389/fmicb.2016.02138 (PMC5225093; doi:10.3389/fmicb.2016.02138)
Supplement: Supplementary file 6 [file Image4.pdf]

Supplementary Material

Knight DR*, Squire MM, Collins DA and Riley TV (2016). Genome analysis of *Clostridium difficile* PCR ribotype 014 lineage in Australian pigs and humans reveals a diverse genetic repertoire and signatures of long-range interspecies transmission. *Front. Microbiol.* **7**:2138. doi: 10.3389/fmicb.2016.02138

*Correspondence: Daniel R. Knight; daniel.knight@uwa.edu.au

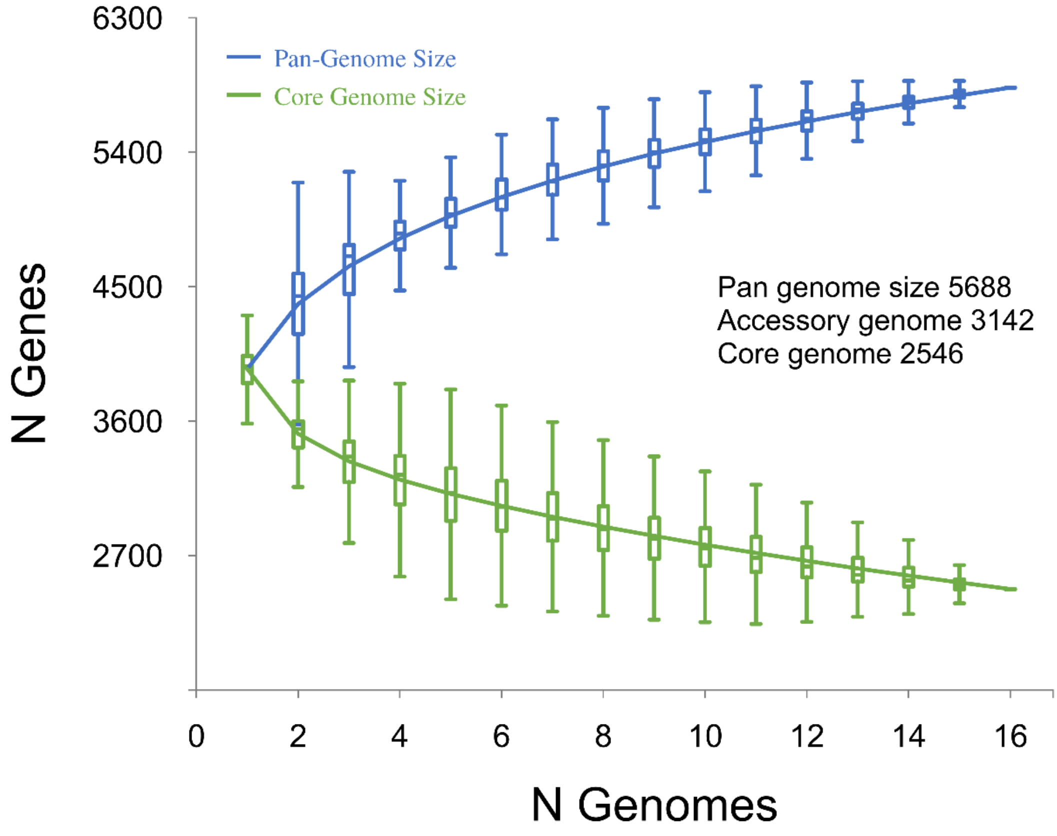

Supplementary Image 4 | Pan-genome analysis of *C. difficile* RT014 from porcine

The total number of genes in the pan (blue) and core (green) genomes from porcine (*n*=16) are plotted as a function of the number of genomes sequentially added (see Methods and manuscript Figure 6 for details).
